# Correlating relative light sensitivity to retinal visual gene expression patterns in three African cichlid fish species

**DOI:** 10.1242/bio.062674

**Published:** 2026-10-12

**Authors:** Gerrit Potkamp, Elena Zhemchuzhnikova, Felicity van Geresteijn, Joyce A. Luijben, Floor A. Munnik, Thomas A. Veldhuis, Martine E. Maan, Louis van de Zande, Rampal S. Etienne

**Affiliations:** Groningen Institute for Evolutionary Life Sciences, University of Groningen, Nijenborgh 7, 9747 AG Groningen, The Netherlands

**Keywords:** Behaviour, Visual pigment, Chromophore, Cyp27c1, Phenotypic plasticity

## Abstract

Visually guided behaviour requires adequate visual sensitivity. Hypotheses on the evolution of vertebrate visual systems largely rely on the assumption that the retinal expression pattern of visual genes is a major determinant of visual sensitivity. However, data on how visual gene expression relates to visual sensitivity and, ultimately, behaviour are equivocal. Here, we correlate relative visual sensitivity to light of different wavelengths with visual gene expression patterns, quantified at various times after the behavioural trials, for three species of African cichlids, raised in different light treatments. To estimate visual sensitivity to different wavelengths, we used the dorsal light response, where fish orient their dorsoventral axis towards the direction of the greatest perceived intensity of incoming light. We found light-treatment-dependent and interspecific variation in relative visual sensitivity. However, at the species and treatment levels, visual gene expression patterns were not indicative of relative visual sensitivity, whereas we did find such correlations at the individual level. Our results indicate that differences in visual sensitivity of cichlid fish cannot be explained by visual gene expression patterns alone, as it may not reflect actual retinal pigment composition and density. In addition, post-retinal (neural) processes might play an important, but as yet unknown, role.

## Teaser

**Summary:** Relative visual sensitivity to different colours of light varies across species and developmental light conditions in African cichlid species; however, these differences cannot be explained by visual gene expression alone.

## INTRODUCTION

There is an extensive body of work that documents interspecific and intraspecific variation in visual system properties at the level of visual pigments on the retina and expression of visual pigment genes in fish (e.g. Bertinetti and Torres-Dowdall, 2025; Carleton and Yourick, 2020; Cortesi et al., 2020; Fuller and Claricoates, 2011; Novales Flamarique et al., 2013; Ricci et al., 2023). Hypotheses regarding the evolutionary implications of such variation make the implicit assumption that expression of visual pigment genes directly translates into visual functioning. However, few studies have actually investigated this link between molecular variation at the pigment gene level and visually guided behaviours (Owens and Rennison, 2017). In the studies that did investigate both molecular and behavioural variation, evidence for a link between visual pigment gene expression and visually guided behaviour is ambiguous (Caves et al., 2025; Fuller et al., 2010; Sakai et al., 2018; Smith et al., 2012; Wilwert, 2022; Wright et al., 2020). Therefore, to understand the evolution of visual pigments, more (experimental) work on this relationship is needed. Here, we quantify behavioural responses to visual cues of different wavelengths in three Lake Victoria cichlid species, in which variation at the level of visually guided behaviours as well as visual pigment gene expression has been documented, to investigate the relationship between the two.

Quantifying the detection of visual cues through behavioural assays is not straightforward. Behaviour is strongly context-dependent, possibly influencing study outcomes. Results of behavioural studies of visual functioning may be influenced by the subjects' social context (e.g. mate choice: Wright et al., 2020) and/or physiological state (e.g. foraging behaviour: Fuller et al., 2010). Optomotor response tests, commonly used to assess visual sensitivity in fish (Maan et al., 2006; Smith et al., 2012), depend on the detection of moving stimuli, in which not all visual pigments may be involved (Krauss and Neumeyer, 2003; Schaerer and Neumeyer, 1996). This context-dependence of complex behaviours may obscure a potential link between visual gene expression and visually guided behaviours, which may explain why several studies did not find such a link (in killifish: Fuller et al., 2010; in cichlids: Wright et al., 2020; Wilwert, 2022; but see Sakai et al., 2016, and Sakai et al., 2018, who did find links between visual gene expression and behaviour in cichlids and guppies, respectively). In the present study, we use the ‘dorsal light response’ to estimate visual sensitivity. This is an evolutionarily highly conserved response of fish in which they orient their dorsoventral axis towards the direction of incoming light (Pfeiffer, 1964). It is hypothesised that this response is a fairly direct result of visual perception (Pfeiffer, 1964); using the dorsal light response to estimate visual sensitivity therefore avoids the context dependence of more complex visual behaviours. The dorsal light response can be used to estimate the relative visual sensitivity to different wavelengths of light by illuminating the fish from both sides with different light stimuli, varying in intensity and spectral composition, and determining the fish dorsoventral axis (Powers, 1978; Silver, 1974). Fish lean towards the stimulus that they perceive as more intense, thereby providing a behavioural readout of relative sensitivity.

We focus on three species of Lake Victoria cichlids (Teleostei: Cichlidae): *Pundamilia* sp. ‘*pundamilia*-like’, *Pundamilia* sp. ‘*nyererei*-like’ and *Haplochromis sauvagei*. Previous studies have shown that both the expression patterns of visual pigment genes and the degree of environmentally induced variation in gene expression differ between these species (Irazábal-González et al., 2024; Wilwert et al., 2023; Wright et al., 2020). We measure both the dorsal light response and visual gene expression patterns in adult fish raised in one of two different developmental light treatments. We hypothesise that if visual gene expression levels are a major determinant of visually guided behaviours, we should observe a correlation with relative light sensitivity measured through the dorsal light response, among species and among groups from different developmental light treatments.

## RESULTS

### Variation in relative sensitivity across species and developmental light treatment groups

We measured the dorsal light response in the Lake Victoria cichlid species *Pundamilia* sp. ‘*pundamilia*-like', *Pundamilia* sp. ‘*nyererei*-like’ and *Haplochromis sauvagei* raised in one of two developmental light treatments (Fig. 1A; see Materials and Methods for details). From these measurements of the dorsal light response, visual sensitivity to blue and red light, relative to green light (Fig. 1B), was estimated. See Materials and Methods below for a detailed description of how the dorsal light response was used to estimate relative sensitivity to blue and red light (see also Fig. 2). All three species showed a dorsal light response. In total, we collected data from 294 trials for the blue test light and 293 trials for the red test light (see Fig. 3A for the data of a single trial and Figs S1, S2 and S3 for the data of all trials). In all trials, the angle of the dorsoventral axis increased (i.e. the fish tilted more towards the test light) with increasing relative intensity of the test light over the control light stimulus: the slope of the response of the dorsoventral axis to relative light intensity was greater than zero for all trials (Fig. 3B). Individual variation in the dorsal light response decreased around relative light intensities closer to the tipping points of the different species raised in the different developmental light treatments (Fig. S4).

**Fig. 1.**
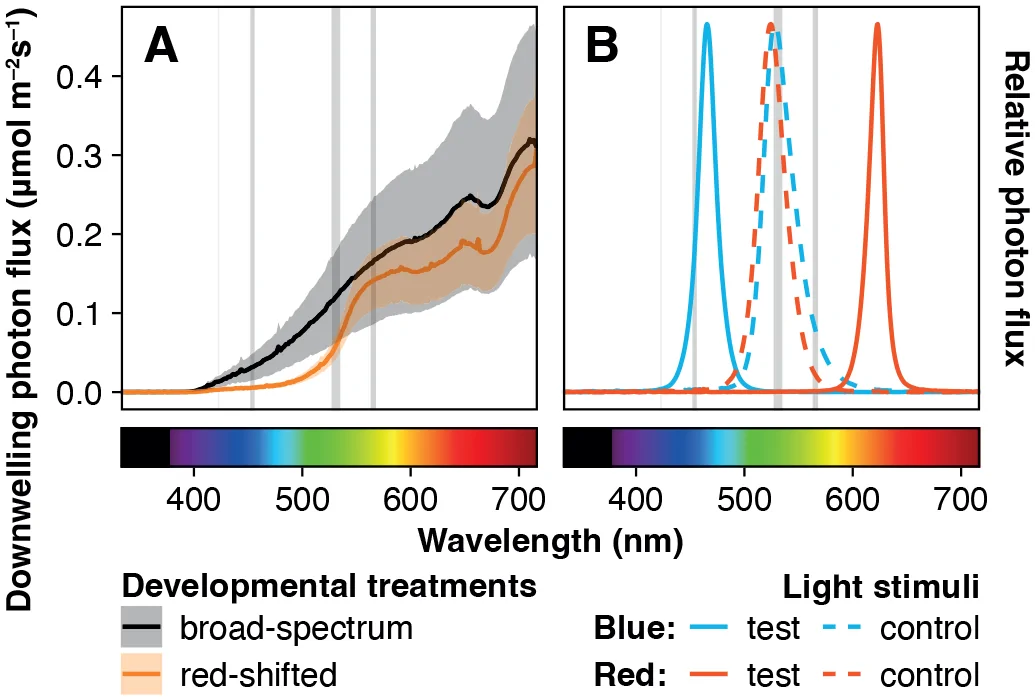
**Spectra of the developmental light treatments and light stimuli used in the experimental set-up.** (A) The two developmental light treatments: broad-spectrum and redshifted light treatments mimicking shallow- and deep-water Lake Victoria habitats, respectively. (B) The spectra of the test and control light stimuli for both the blue and red test lights. Vertical bands denote the peak absorbances of the four opsins (in pigments bound to a vitamin-A_1_-derived chromophore) expressed by the three species included in this study (from left to right: *sws2b*, *sws2a*, *rh2a*, and *lws*, based on Carleton, 2009; Spady et al., 2006). Absorption spectra for pigments bound to vitamin-A_1_-derived chromophore shift ∼4 nm (*sws2b*) to ∼45 nm (*lws*) towards longer wavelengths. Bands around developmental spectra represent one standard error. Spectra for the control stimuli differ slightly for the blue and red test lights due to the use of different coloured filters (used to prevent reflection of light; see also Fig. 2 and Table S1).

**Fig. 2.**
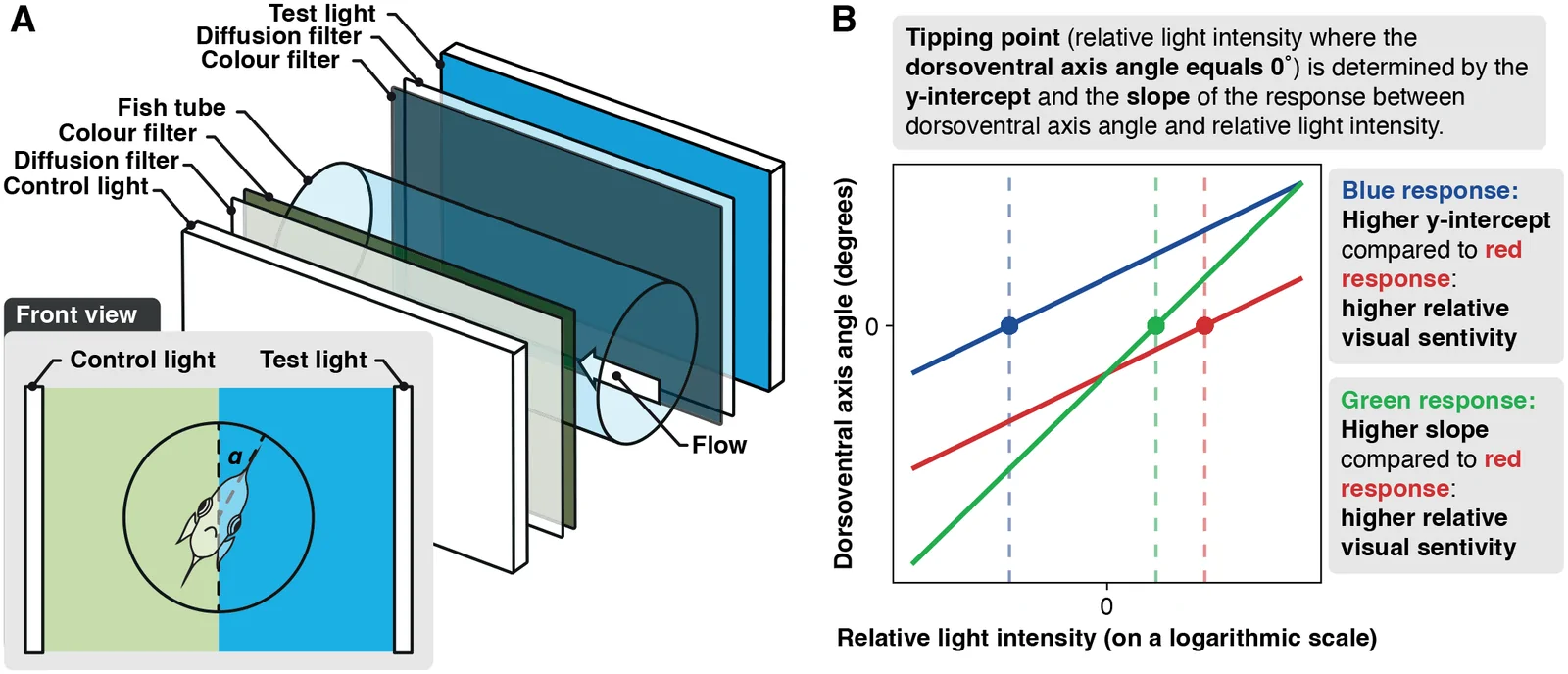
**Schematic overview of the experimental set-up to measure the dorsoventral axis angle in response to two light stimuli and the relation between variation in dorsoventral light response and the tipping point.** (A) An individual fish is kept in a transparent tube and illuminated with a test and control light stimulus, one from each side. A diffusion filter and colour filter were placed between the light source and the fish (Table S1), and a flow was directed through the tube to orient the fish in such a way that one light stimulus illuminated each eye. The angle of the dorsoventral axis (*α*) was measured using a webcam in front of the tube, as shown in the front view panel. (B) Tipping points are marked with a dashed line (i.e. the relative intensity at which the dorsoventral axis angle equals 0°).

**Fig. 3.**
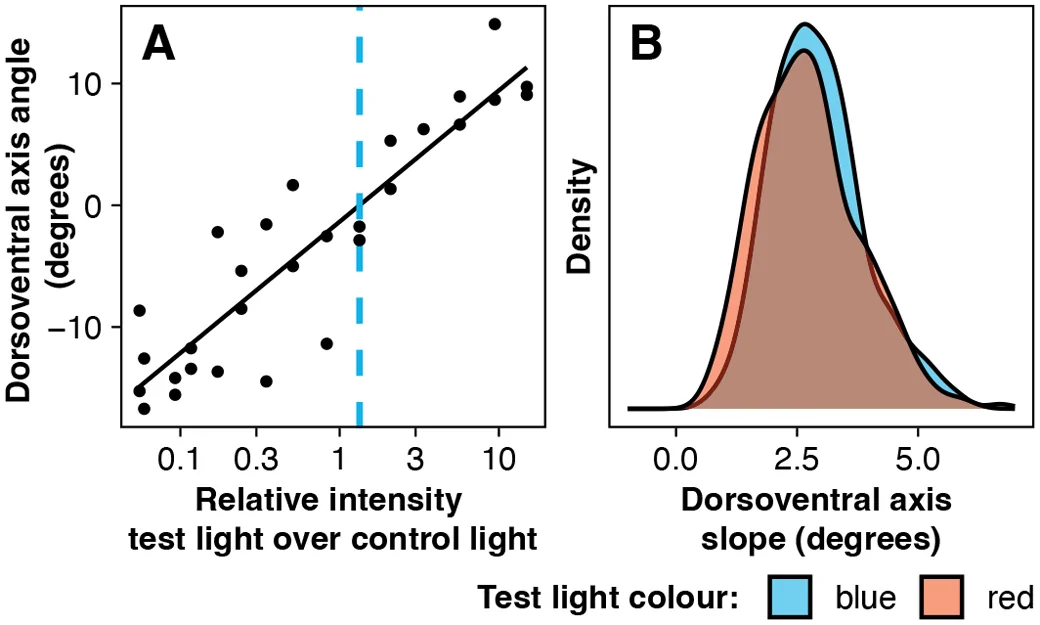
**Dependence of the dorsoventral axis angle on the relative intensity of light stimuli.** (A) The angle of the dorsoventral axis of one individual in a single trial, when presented with a range of different relative intensities (on a logarithmic scale) of test light over control light. (B) The distribution of the slopes of the linear relationships between the angle of the dorsoventral axis and the relative light intensity (as shown in A) for all trials (*n=*294 for the blue test light, *n=*293 for the red test light). The relative intensity at which the dorsoventral axis angle equals 0° is marked with a dashed line (A); slopes of the linear relationships (B) are calculated based on the random effects estimated in the linear mixed-effects models for relative blue- and red-light sensitivity.

Relative blue-light sensitivity differed significantly between *Pundamilia* sp. ‘*nyererei*-like’ individuals from different developmental light treatments (Figs 4 and 5). In *Pundamilia* sp. ‘*nyererei*-like’ from the broad-spectrum developmental light treatment, the dorsoventral axis angle under the blue test light was significantly larger (i.e. fish tilted more towards the blue test light or less towards the green control light) compared to fish from the redshifted developmental light treatment, across all relative light intensities (*z*-ratio=2.15; *P=*0.031), which resulted in a significantly different tipping point (*z*-ratio=2.54; *P=*0.033; Figs 4 and 5). In *Pundamilia* sp. ‘*pundamilia*-like’, we found no difference in relative sensitivity to blue light. Fish raised in the broad-spectrum developmental light treatment had a significantly steeper slope for the response of the dorsoventral axis angle to the relative light intensity than did fish from the redshifted light treatment (*z*-ratio=1.97; *P=*0.049; Fig. S5), but this did not result in a significantly different tipping point (*z*-ratio=0.40; *P=*0.396; Figs 4 and 5). We found no effect of developmental light treatment on blue-light sensitivity in *H. sauvagei*. For relative red-light sensitivity, no significant effects of developmental light treatment were found in any of the three species (Table S2).

**Fig. 4.**
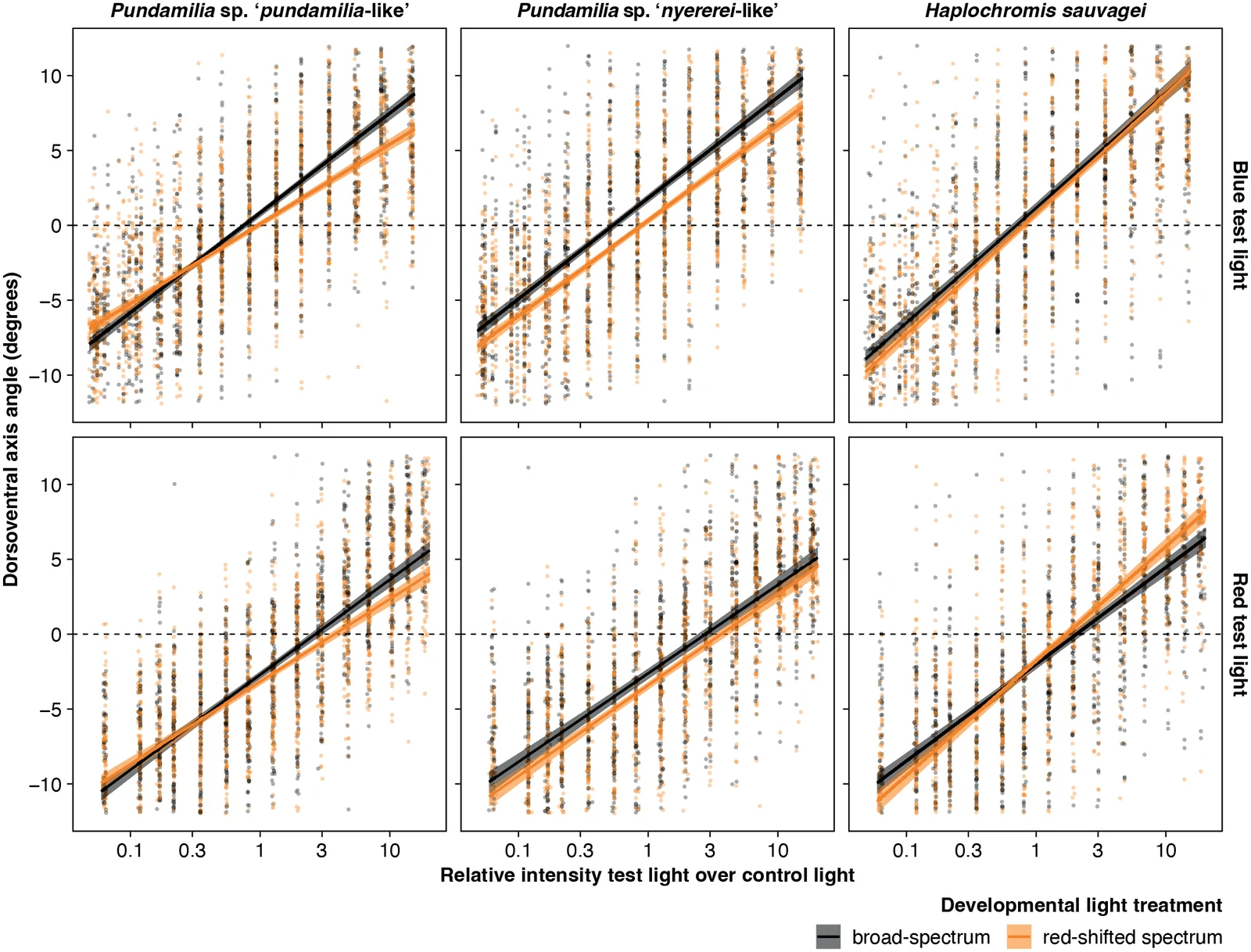
**Dorsoventral axis angle across relative intensities of blue-light (top panels) and red-light (bottom panels) stimuli in fish raised in different developmental light treatments.** Confidence bands are based on bootstrapping a full model (*n*=10,000; bands contain 95% of bootstrapped estimates). Some observations fall outside the dorsoventral axis angle scale; for all data points, see Fig. S3.

**Fig. 5.**
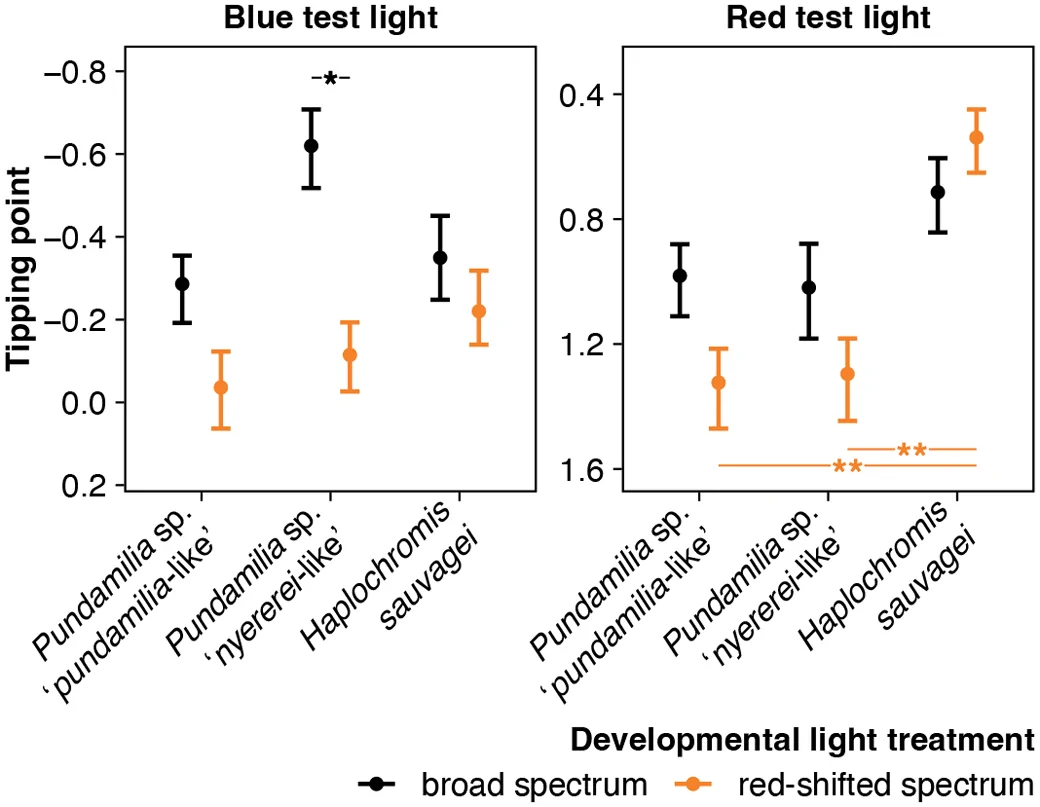
**Predicted developmental light and species effect on the tipping point for blue and red test lights, estimated using coefficients of the linear mixed-effects model.** Effects are estimated for fish with a median age and for median control light intensities. Error bars are based on bootstrapping (*n*=10,000; error bars contain 95% of bootstrapped estimates). Contrasts between groups: *P<0.05; **P<0.01. Note that the tipping-point axis is reversed, with lower values corresponding to higher relative light intensities, and that species contrasts are specific to different developmental light treatments.

Relative sensitivity to blue light did not differ significantly between species. We did detect significant differences in relative sensitivity to red light between species (Figs 4 and 5). The intercept of the response of the dorsoventral axis was significantly higher in *H. sauvagei* compared to *Pundamilia* sp. ‘*nyererei*-like’ from the redshifted treatment (*z*-ratio=2.41; *P=*0.043), and both the intercept and the slope of the response differed between *H. sauvagei* and *Pundamilia* sp. ‘*pundamilia*-like’ from the redshifted treatment (intercept: *z*-ratio=2.48; *P=*0.036; slope: *z*-ratio=3.00; *P=*0.008). These differences resulted in a significantly different tipping point between *H. sauvagei* and both *Pundamilia* sp. ‘*nyererei*-like’ (*z*-ratio=3.43; *P=*0.004) and *Pundamilia* sp. ‘*pundamilia*-like’ (*z*-ratio=3.35; *P=*0.004) from the redshifted treatment (Fig. 5). We did not find a significant species difference in fish from the broad-spectrum developmental treatment (Table S2).

We found that the dorsoventral axis angle under the blue test light increased significantly with age in *Pundamilia* sp. ‘*nyererei*-like’ (−9.8×10^−4^° change per day; 95% confidence interval: −3.9×10^−3^° to −8.6×10^−5^° per day), indicating that relative sensitivity to blue light decreases with age in *Pundamilia* sp. ‘*nyererei*-like’. We found no significant age effects in the other species or in the relative sensitivity to red light.

The absolute intensity of the control light did not have a significant effect on the dorsoventral axis angle under the blue-test-light stimulus (−0.03° change in dorsoventral axis angle per μmol photons m^−2^ s^−1^; 95% confidence interval −0.07° to 0.01° per μmol photons m^−2^ s^−1^). We did find a small but statistically significant effect of absolute intensity of the control light for the red-test-light stimulus (−0.31° change in dorsoventral axis angle per μmol photons m^−2^ s^−1^; 95% confidence interval −0.38° to −0.24° per μmol photons m^−2^ s^−1^); however, this effect was substantially smaller than the effect of the relative intensity of the test over control light (3.2° difference in dorsoventral axis angle over the range of control light intensity, compared to 14.0–19.4° over the range of relative intensities, depending on the experimental group), indicating that the dorsoventral axis angle is mostly determined by the relative intensity of the test over control light rather than the absolute intensity of light stimuli for both the blue- and red-test-light stimuli.

### Variation in visual gene expression

We found a significantly higher ratio of *sws2ab* (sum of expression of *sws2a* and *sws2b*) to *rh2a* expression in *Pundamilia* sp. ‘*nyererei*-like’ from the broad-spectrum light treatment compared to the redshifted treatment (*t*-ratio=2.15; d.f.*=*78.6; *P=*0.035). In the other two species, no significant effect of developmental light treatment was found (Fig. 6A).

**Fig. 6.**
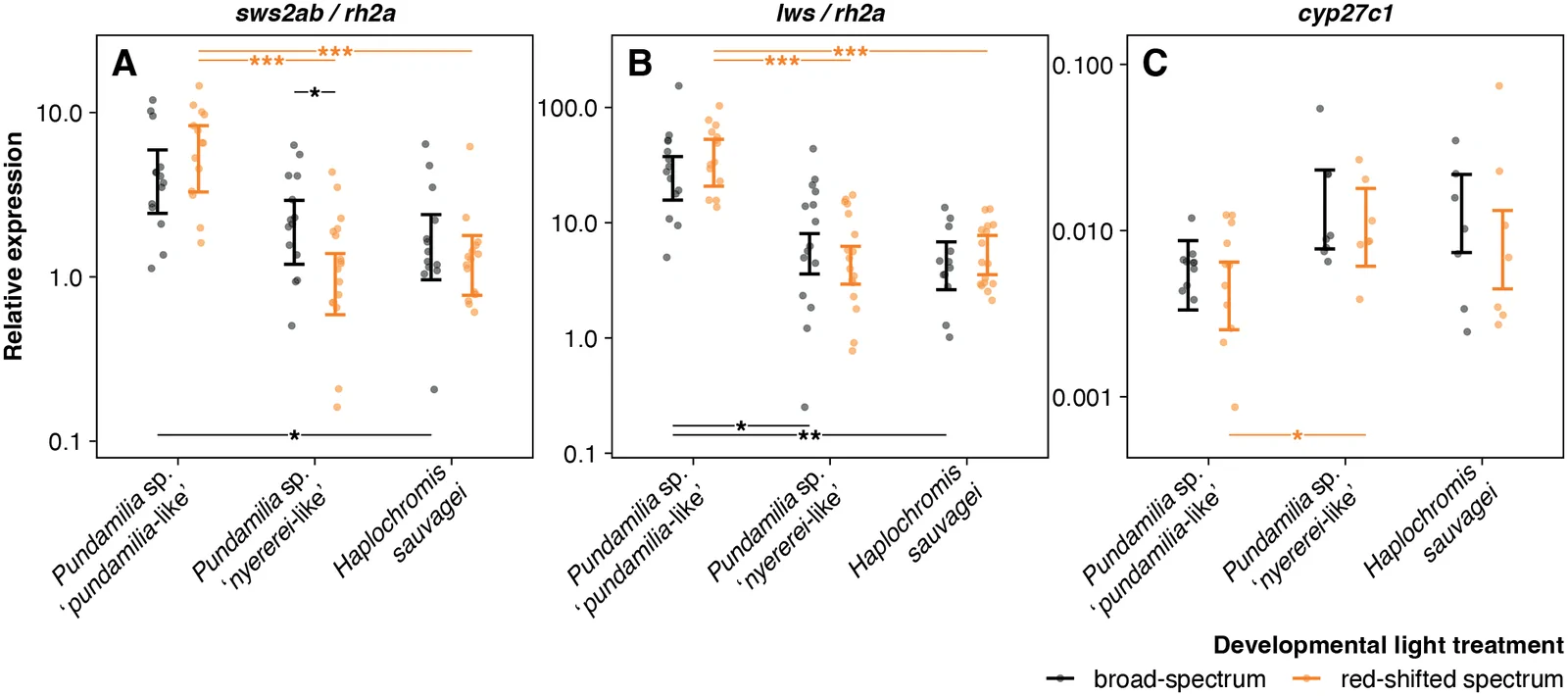
**Variation in opsin expression ratio (A,B) and *cyp27c1* expression (C) in fish raised in different developmental light treatments.** Error bars denote two standard errors (estimated for a median age and delay between behavioural trials and retinal sampling); symbols denote individual observations. Contrasts: *P<0.05; **P<0.01; ***P<0.001. Note that species contrasts are specific to different developmental light treatments.

The *sws2ab*-to-*rh2a* expression ratio differed significantly between species as well. It was significantly higher in *Pundamilia* sp. ‘*pundamilia*-like’ than in both *Pundamilia* sp. ‘*nyererei*-like’ (but only in fish from the redshifted spectrum; *t*-ratio=5.37; d.f.*=*94.9; *P<*0.0001) and *H. sauvagei* (in fish from both developmental treatments; broad spectrum: *t*-ratio=2.47; d.f.*=*90.6; *P=*0.041; redshifted: *t*-ratio=4.96; d.f.*=*93.4; *P<*0.0001). No significant differences were found between *Pundamilia* sp. ‘*nyererei*-like' and *H. sauvagei* (Table S3).

We did not find a treatment effect in the expression ratio of *lws* to *rh2a* (Fig. 6B). There was a significant species effect in this expression ratio: the ratio of *lws* to *rh2a* was significantly higher in *Pundamilia* sp. ‘*pundamilia*-like’ than in both *Pundamilia* sp. ‘*nyererei*-like' (broad-spectrum individuals: *t*-ratio=2.88; d.f.*=*89.9; *P=*0.014; redshifted individuals: *t*-ratio=4.03; d.f.*=*84.6; *P=*0.0004) and *H. sauvagei* (broad spectrum: *t*-ratio=3.69; d.f.*=*93.8; *P=*0.001; redshifted: *t*-ratio=4.34; d.f.*=*97.5; *P=*0.0001) but did not differ significantly between *Pundamilia* sp. ‘*nyererei*-like' and *H. sauvagei* (Table S3).

The relative expression of *cyp27c1* did not differ between developmental treatments (Table S3). We did find that the relative expression of *cyp27c1* differed significantly between species (Fig. 6C): *cyp27c1* expression was significantly higher in *Pundamilia* sp. ‘*nyererei*-like’ than in *Pundamilia* sp. ‘*pundamilia*-like’ (but only in fish from the redshifted spectrum: *t*-ratio=2.61; d.f.*=*69.8; *P=*0.029).

We detected several age effects in visual gene expression patterns (Fig. S6). In *Pundamilia* sp. ‘*nyererei*-like’, both the *sws2ab*-to-*rh2a* expression ratio and the *lws*-to-*rh2a* expression ratio increased significantly with age (*sws2ab* to *rh2a*: the expression ratio, transformed with the natural logarithm, changed 8.1×10^−4^ units per day of age; 95% confidence interval: 9.7×10^−5^ to 1.5×10^−3^ per day; *lws* to *rh2a*: 1.6×10^−3^ units per day of age; 95% confidence interval: 7.7×10^−4^ to 2.5×10^−3^ per day). In *H. sauvagei*, the relative expression of *cyp27c1* decreased with age (−4.6×10^−3^ units per day of age; 95% confidence interval: −8.5×10^−3^ to −7.0×10^−4^ per day).

Patterns of visual gene expression generally did not change with increasing time between behavioural trials and retinal sampling (Fig. S7). Only in *Pundamilia* sp. ‘*pundamilia*-like’ from the redshifted treatment did we find that *cyp27c1* expression increased with an increasing delay between behavioural trials and retinal sampling. In the other fish, we found no significant effects of sampling delay on visual gene expression.

### Relating variation in relative sensitivity to visual gene expression at the group level

At the group level, interspecific and developmental light-induced differences in visual gene expression did not fully align with the observed differences in relative sensitivity. At the interspecific level, the differences observed in the expression ratio of *sws2ab* to *rh2a* (Fig. 6) were not reflected in tipping points for blue light, in which no interspecific differences were detected (Fig. 5). For relative sensitivity to red light, interspecific differences in tipping points between *H. sauvagei* and both *Pundamilia* species (Fig. 5) contrasted with the patterns observed in the relative expression of *lws* to *rh2a*, where *Pundamilia* sp. ‘*pundamilia*-like’ differed from the two other species (Fig. 6). In general, tipping points for both blue and red lights of both *Pundamilia* species were similar, and they were different from the tipping points observed in *H. sauvagei* (Fig. 5), while in visual gene expression patterns, *Pundamilia* sp. ‘*pundamilia*-like’ differed from the two other species, which were more similar (Fig. 6). The absence of a correlation between interspecific variation in gene expression and the tipping points cannot be explained by the delay between trials and retinal sampling because there was a general lack of a trend in visual gene expression with the delay between trials and retinal sampling Fig. S7. Group-level variation in the slope for the response of the dorsoventral axis angles to relative light intensity did not align with visual gene expression patterns either (Fig. S5).

Treatment differences in relative sensitivity did, at the group level, partly align with differences in relative sensitivity. We detected only treatment effects in relative blue-light sensitivity in *Pundamilia* sp. ‘*nyererei*-like’, where the difference in tipping point under the blue test light (Fig. 5) corresponded at the group level to a higher expression ratio of *sws2ab* to *rh2a*, which was the only statistically significant treatment effect in visual gene expression patterns (Fig. 6).

With the inclusion of visual gene expression patterns in the statistical models, the statistically significant treatment and species effects observed in dorsoventral axis angle under the blue test light were no longer statistically significant (Table S4), suggesting that these differences could at least partly be explained by variation in gene expression patterns. For red-light sensitivity, we still found a statistically significant species effect despite the inclusion of gene expression patterns: the dorsoventral axis under the red test light in individuals from the redshifted treatment was significantly higher in *H. sauvagei* than in *Pundamilia* sp. ‘*pundamilia*-like’ (*z*-ratio=2.56; *P=*0.028), resulting in a significantly different tipping point between the species (*z*-ratio=3.09; *P=*0.012). This indicates that the differences in relative sensitivity to red light between these species could not be fully explained by gene expression patterns.

### Relating variation in relative sensitivity to visual gene expression at the individual level

While visual gene expression did not fully explain between-group variation in relative sensitivity to blue- and red-light stimuli, we did find that opsin gene expression covaried with some of the variation in relative light sensitivity across individuals (Fig. 7).

**Fig. 7.**
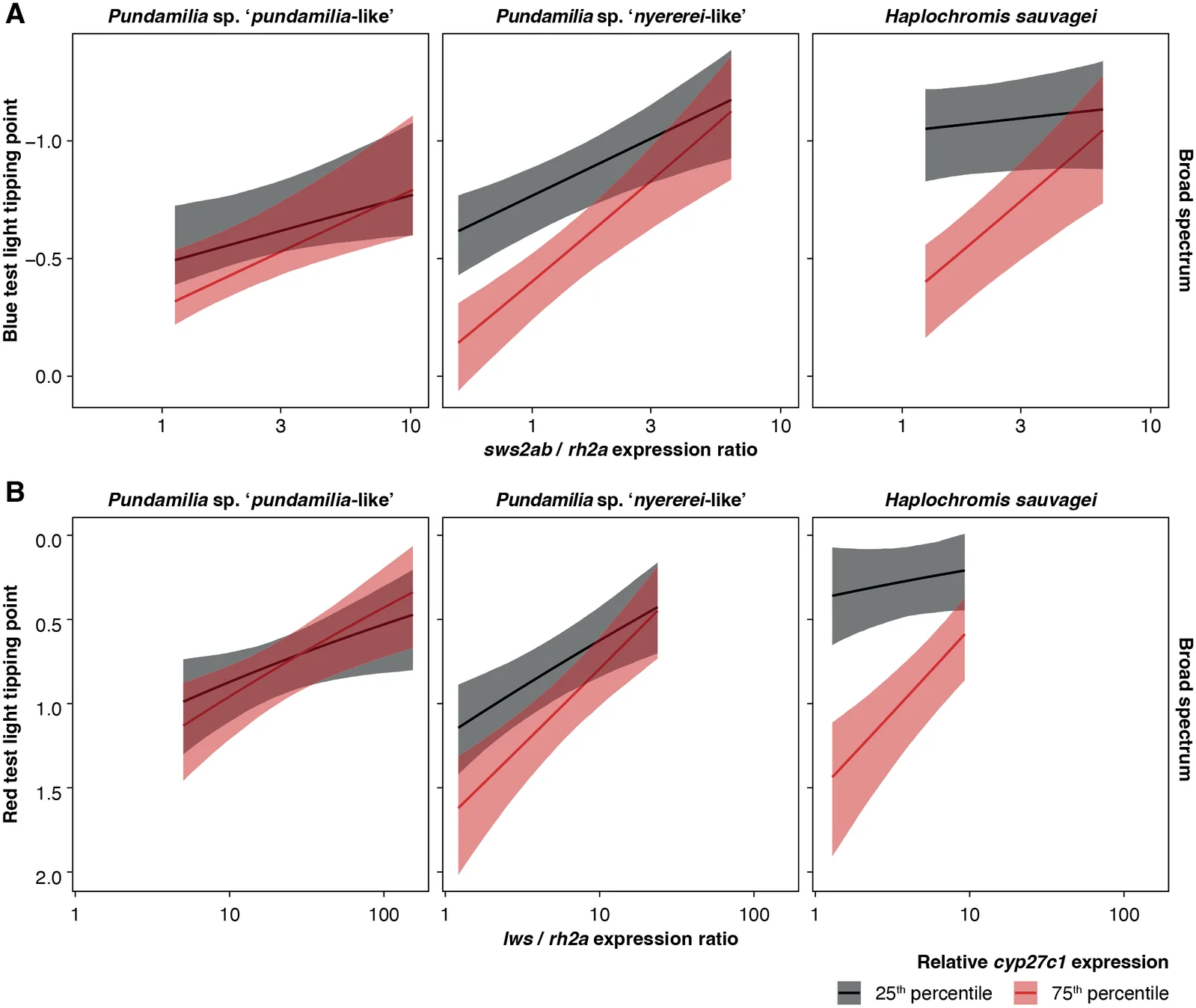
**Estimated relation between tipping points for blue (A) and red (B) test lights and opsin gene expression.** Predictions are shown for different levels of *cyp27c1* expression. The relation is estimated for a sampling delay of 7 days, using estimated model coefficients for fish from the broad-spectrum treatment and for a median control light intensity and age. Confidence bands are based on bootstrapping estimates (*n*=10,000; bands contain 95% of bootstrapped estimates). Note that the tipping-point axis is reversed, with lower values corresponding to higher relative light intensities.

For both the blue and red test lights, the dorsoventral axis angle tended to increase with the *sws2ab*-to-*rh2a* and the *lws*-to-*rh2a* expression ratios, respectively.

The relationship between the *lws*-to-*rh2a* expression ratio and relative red light sensitivity was, based on the model predictions, stronger for individuals with a higher *cyp27c1* expression. This resulted, for *H. sauvagei* and *Pundamilia* sp. ‘*nyererei*-like’ with a high *cyp27c1* expression (*cyp27c1* expression at the 75th percentile), in a significantly increased tipping point (i.e. an increased relative sensitivity to red light) in fish with a high *lws*-to-*rh2a* expression ratio compared to fish with a low expression ratio (Fig. 7; Fig. S8). In other words, the relative sensitivity to red light increased with the *lws*-to-*rh2a* expression ratio in *H. sauvagei* and *Pundamilia* sp. ‘*nyererei*-like’ individuals with high *cyp27c1* expression levels. We observed the same trends for the other groups of fish (fish with low *cyp27c1* expression, individuals from *Pundamilia* sp. ‘*pundamilia*-like’), for both the relationship between red-light sensitivity and the *lws*-to-*rh2a* expression ratio and the relationship between blue-light sensitivity and the *sws2ab*-to-*rh2a* expression ratio, but those trends were not statistically significant (Fig. 7; Fig. S8). We found no correlation between the slope for the response of the dorsoventral axis angles to relative light intensity and the *sws2ab*-to-*rh2a* or the *lws*-to-*rh2a* expression ratios (Fig. S9).

The observed individual-level correlations between the opsin expression ratio and dorsoventral axis angle were strongest for individuals with a short delay between behavioural trials and sampling of the retinal tissue (Fig. S10). With an increasing sampling delay, the strength of the relationship between *sws2ab*-to-*rh2a* and *lws*-to-*rh2a* expression ratios and blue- and red-light sensitivity, respectively, decreased.

The relative expression of *cyp27c1* was negatively correlated with relative sensitivity to both relative blue- and red-light sensitivity (Fig. S11). The significance of this correlation varied across species, developmental treatments, opsin expression ratio and the sampling delay, but we found significant decreases in tipping point for blue light with higher *cyp27c1* expression in *Pundamilia* sp. ‘*nyererei*-like’ and for red light in *Pundamilia* sp. ‘*nyererei*-like’ and *H. sauvagei* (Fig. S12). We found no correlation between the slope of the response of the dorsoventral axis angles to relative light intensity and *cyp27c1* expression (Fig. S13).

## DISCUSSION

Visually guided behaviour is dependent on the functioning of the visual system. Studies on the (adaptive) evolution of the cichlid visual system predominantly focus on the level of visual gene sequence and expression (e.g. Bertinetti and Torres-Dowdall, 2025; Carleton and Yourick, 2020; Carleton et al., 2016; Nandamuri et al., 2017; Ricci et al., 2023; Wilwert et al., 2022) and rely on the assumption that the expression pattern of visual pigment genes is a major determinant of the visual sensitivity of the organism. Here, we tested this assumption by determining relative sensitivity to different light stimuli and assessing whether this is correlated with variation in relative expression levels of visual genes. We used the dorsal light response to quantify visual sensitivity. In contrast to optomotor assays, this method does not involve detection of moving stimuli, potentially providing a less biased representation of colour perception (Kaiser and Liske, 1974; Krauss and Neumeyer, 2003; Schaerer and Neumeyer, 1996; Smith et al., 2012). To the best of our knowledge, the dorsal light response has not been used previously to assess a possible link between gene expression patterns and behaviour. At the individual level, we found correlations between relative sensitivity and gene expression levels. However, interspecific and, to a lesser extent, light-treatment-induced variation in relative sensitivity (i.e. group-level variation) was only partly explained by variation in gene expression levels. This discrepancy suggests that the assumption that visual gene expression patterns are a major determinant of across-group variation in visual sensitivity needs reconsideration, although gene expression data that are time matched to the behavioural trials (instead of data with a delay between behavioural trials and retinal sampling) are needed to firmly establish this conclusion.

Evidence for correlations between visual gene expression levels and visually guided behaviour at the individual level is ambiguous. Correlations have been found in Lake Malawi cichlids (Smith et al., 2012) and in guppies (Sakai et al., 2016, 2018). However, the absence of correlations has been reported as well, for instance, in Lake Victoria cichlids (habitat choice: Wilwert, 2022; female mate choice: Wright et al., 2020), as well as in other fish (pecking preference of different coloured objects in killifish: Johnson et al., 2013). This discrepancy can be partly explained by the fact that these are complex behaviours, several steps downstream of the actual detection of the visual cues (Caves et al., 2025). Our study also indicates that a more primary behavioural response to different light cues cannot be fully explained by visual gene expression.

Our results suggest that post-photoreceptor processes play a role in the visual sensitivity difference between the species included in this study. Such processes can relate to variation in the phototransduction pathway (the process that converts photons absorbed by visual pigments into a neural response; Carleton and Yourick, 2020; Carleton et al., 2016; Shimmura et al., 2017). In addition, pigment density and/or retinal surface differences may play a role in the registration capacity for an initial light signal, but how expression of opsin genes relates to the actual distribution and density of visual pigments on the retina is still unclear (see Dalton et al., 2017).

Another factor may be the downstream processing of signals in the brain. All visual cues start with the absorption of photons of a specific wavelength on the retina. If we consider the downstream processing of this visual signal a Markov chain, then information on colour and intensity of the signal can be lost, and lost information cannot be regained (the data processing inequality). Nevertheless, in complex biological systems, neural interactions with other systems may modify initially received external signals. For example, retinal ganglion cells in mice – which are post-synaptic partners of photoreceptor neurons – have been identified as releasing inhibitory neurotransmitters that dampen behavioral sensitivity to light (Sonoda et al., 2020). This shows that the signal level can be amplified or dampened by nervous processes downstream of a photoreceptor neuron. While various visual processing pathways have been identified and studied in fish (e.g. Baier and Scott, 2024; Bollmann, 2019; Vanegas and Ito, 1983), including in cichlids (Calvo et al., 2023), still very little is known about (interspecific variation in) the processing of visual signals in cichlids. Studying variation in visual processing pathways across species could help in understanding variation in visual functioning and the evolutionary implications of this variation.

We did not measure the expression of the rod opsin rhodopsin (*rh1*), involved in dim-light vision. *rh1* absorbs light in the green part of the visual spectrum (Carleton et al., 2020). Interspecific variation in *rh1* expression has been described in Lake Tanganyika species, although this variation is less than that observed for opsins involved in colour vision and is mostly associated with large differences in light intensity between shallow- and deep-water habitats (Ricci et al., 2023). Variation in *rh1* expression could affect visual sensitivity to green light (a higher expression of *rh1* may result in a higher sensitivity to green light, especially in low-environmental-light conditions); however, since fish were kept light adapted during the experimental trials, the effect of rh1 is expected to be limited.

With our study design, we cannot rule out that the behavioural testing itself affected visual gene expression patterns in the short or the long term. Wilwert (2022) documented some differences in opsin expression patterns between fish that were subjected to behavioural trials and fish that were not. The expression patterns measured after the behavioural tests may therefore not accurately reflect the expression patterns during behavioural testing. In our study, we used a minimum delay of 5 days between behavioural trials and sampling to minimise the impact of potential short-term effects on gene expression and corrected for sampling delay in our statistical modelling to account for long-term effects. To truly understand the relation between visual gene expression patterns and relative sensitivity, more animals would be needed, including animals that did not participate in behavioural trials, to create a more controlled sampling design. With an increased sample size, the individual-level correlations between gene expression patterns and relative sensitivity, of which we found some evidence here, may become more evident.

For some of the individuals in our study, sampling delay was considerably longer than 5 days. The strength of individual-level correlations between relative sensitivity and visual gene expression patterns decreased with sampling delay, which could explain why only weak individual-level correlations were found. Retinal sampling shortly after the behavioural trials would be needed to firmly establish the individual-level correlations. It is, however, unlikely that the sampling delay in this study fully obscured group-level effects. We found that visual gene expression patterns are, at the group level, relatively stable over time (although we did see some age effects) and did not change substantially with an increasing sampling delay (see also Irazábal-González et al., 2024; Wilwert et al., 2023), indicating that there were no large long-term effects of the behavioural trial on visual gene expression patterns. The expression patterns found in the current study are quantitatively similar to group-level gene expression patterns described earlier for these species, both in laboratory conditions and in the wild, where, in general, expression patterns of *Pundamilia* sp. ‘*nyererei*-like’ are similar to *H. sauvagei*, while *Pundamilia* sp. ‘*pundamilia*-like’ differs from those two species, with, for example, a higher relative *lws* and lower *cyp27c1* expression levels (Irazábal-González et al., 2024; Wilwert et al., 2022, 2023; Wright et al., 2019, 2020). In contrast, the behavioural data presented in this study showed that relative sensitivities of both *Pundamilia* species were similar, and *H. sauvagei* had relative sensitivities deviating from *Pundamilia* spp., a pattern never observed in visual gene expression studies before. However, to firmly establish our conclusion that visual gene expression does not explain group-level differences in visual sensitivity, more time-matched gene expression data are needed.

The relation between *cyp27c1* expression and visual sensitivity was not straightforward. We found that the relation between relative red-light sensitivity and the *lws*-to-*rh2a* expression ratio was stronger in fish with higher *cyp27c1* expression. This is consistent with the hypothesised function of cyp27c1, mediating a switch from vitamin-A_1_- to vitamin-A_2_-derived chromophores, which results in a shift in absorption to longer wavelengths (Parry and Bowmaker, 2000). This shift has a much larger effect on long-wavelength-absorbing pigments than on short-wavelength-absorbing pigments (Carleton et al., 2016). Thus, a change in the relation between relative red-to-green sensitivity and the *lws*-to-*rh2a* expression ratio, as quantified here in individuals with higher *cyp27c1* expression, can be expected. However, expression of *cyp27c1* was, at the individual level, negatively correlated with relative sensitivity to both blue and red lights (though not always statistically significant), suggesting a more complicated effect of cyp27c1 on colour sensitivity. In cichlids, only a weak correlation between the chromophore ratio and *cyp27c1* expression has so far been documented, based on a few neotropical cichlid species (Torres-Dowdall et al., 2017). A better understanding of the dynamics of chromophore usage in cichlids, and the exact role of cyp27c1 in those dynamics, is therefore needed to be able to assess its potential role in determining variation in visual functioning (Escobar-Camacho et al., 2019; Wilwert et al., 2022, 2023).

There was an effect of developmental light treatments on relative sensitivity to blue-light stimuli. Fish raised in the redshifted developmental light treatment, which lacks short-wavelength (blue) light, had lower relative sensitivity to the blue-light stimulus, compared to fish raised in the broad-spectrum treatment. This ‘wavelength matching’ (Fuller et al., 2004) has been documented in several vertebrate taxa, at the level of behaviour (cichlids: Smith et al., 2012), at the level of visual gene expression (cichlids: Hofmann et al., 2010; Nandamuri et al., 2017) and as variation in neural responses (lizards: Leal and Fleishman, 2002; stickleback: McDonald and Hawryshyn, 1995). Such plastic responses may allow individuals to quickly adapt to local conditions, which can play a role in evolutionary adaptation (Levis and Pfennig, 2021).

Variation in visual gene expression patterns between species and individuals is widely observed across vertebrate taxa, particularly in fish (Bloch et al., 2015; Fuller and Claricoates, 2011; Fuller et al., 2004; Gisbert et al., 2020; Härer et al., 2018; Hofmann et al., 2010; Nandamuri et al., 2017; Novales Flamarique et al., 2013; O'Quin et al., 2010; Schott et al., 2022; Smith et al., 2011; Wilwert et al., 2022, 2023; Wright et al., 2019, 2020). Our results show that visual gene expression does not necessarily explain variation in visual sensitivity and downstream complex visually guided behaviours. Future work should address other aspects of the visual pathway and study the relative importance of pigment versus non-pigment-related components.

## MATERIALS AND METHODS

### General approach

To estimate relative light sensitivity, we measured the dorsal light response to blue and red lights (relative to green) in a heterogeneously aged group of adult males and females of three species of African cichlid fish, raised in different light treatments. After behavioural testing, fish were sacrificed to measure visual gene expression levels. Unfortunately, the delay between testing and expression measurement varied greatly between individuals (from 1 to 36 weeks; median 4 weeks). This was because the same individuals were planned to be involved in additional behavioural experiments (which eventually did not take place). While not ideal, this variation was accounted for in the statistical analyses. We first assessed the effect of developmental light treatments and species identity on the dorsal light response. Then we analysed the relationship between behavioural response and gene expression. This study was conducted under the approval of the Institutional Animal Care and Use Committee of the University of Groningen (licence number AVD105002016464).

### Using the dorsal light response to estimate visual sensitivity

We estimated relative visual sensitivity to differently colored light stimuli using the fishes’ dorsal light response, whereby fish orient their dorsoventral axis toward the direction of incoming light (first studied by Von Holst, 1935, in the wrasse *Symphodus rostratus*; reviewed by Pfeiffer, 1964). The dorsal light response represents the optic component of the mechanism by which fish orient their body in the water column. Information from the direction of light is integrated with information from the utriculus (representing the static component of the orientation mechanism, detecting gravity) in the central nervous system. The optic component in this mechanism is hypothesised to correct the orientation of the fish in, for example, turbulent waters, where the static component might misinform the fish (Pfeiffer, 1964). If the information from the static component does not align with information from the optic component, for example when the fish is illuminated from one side, the fish will orient its dorsoventral axis in-between the direction of gravity and the incidence of light (Pfeiffer, 1964). This dorsal light response is exhibited by most free-swimming fish species, including cichlids (Bogenschütz, 1961; Canella, 1937; McCormack and McDonnell, 1994; Silver, 1974; Takeuchi and Endo, 2008; Von Holst, 1950), and is mediated by the fish visual system (Powers, 1978).

The dorsal light response can be used to estimate the relative visual sensitivity to different wavelengths of light by exposing an individual to two different light stimuli at once, one on each side (Fig. 2A; Fig. S14; Douglas and Hawryshyn, 1990; Lang, 1967; Muntz et al., 1996; Powers, 1978; Silver, 1974; Thibault, 1949). Such exposure will result in the fish tilting its dorsum towards the light stimulus that it perceives as most intense. The degree of tilt relates to the difference in perceived intensity: a larger difference results in a stronger tilt (Brodsky, 1999). The relative intensity at which the dorsoventral axis angle equals 0° – that is, when the fish does not tilt toward either the test or control light and is therefore assumed to perceive both stimuli as equally intense – serves as a measure of the fish's visual sensitivity to the test light relative to the control light (Muntz et al., 1996; Powers, 1978; Silver, 1974). A lower relative intensity at which the dorsoventral axis equals 0° corresponds to a higher sensitivity to the test light relative to the control light: the fish perceives both stimuli as equally intense for a lower intensity of the test light.

The strength of the dorsoventral light response can depend on external factors or the physiological state of the fish (i.e. the angle at which a fish tilts towards incoming light can vary depending on external factors; Brodsky, 1999; Pfeiffer and Riegelbauer, 1978; Von Holst, 1948). This may affect the dorsoventral axis angles when the test and control light stimuli are not perceived as equally intense. By using the relative intensity at which the dorsoventral axis angle equals 0° as a measure for relative visual sensitivity, we minimise the effect of individual variation in the strength of the dorsal light response. We will estimate this point from statistical models that describe the dorsoventral axis angle as a function of relative light intensity and will refer to the relative intensity where the dorsoventral axis angle equals 0° as the ‘tipping point’.

We analysed the dorsal light response in linear mixed-effects models, predicting the dorsoventral axis angles of the different species and fish raised in different developmental light treatments as a function of relative light intensity. We used the slope and intercept for the response of the dorsoventral axis angle to relative light intensity as predicted by the linear mixed-effects models to estimate the tipping point (Fig. 2B).

Here, we quantified the dorsal light response to blue and red lights relative to green light in the endemic Lake Victoria species *Pundamilia* sp. ‘*pundamilia*-like’, *Pundamilia* sp. ‘*nyererei*-like’ and *H. sauvagei*. *Pundamilia* sp. ‘*pundamilia*-like’ is a shallow-water species from the murky Mwanza Gulf, while *Pundamilia* sp. ‘*nyererei*-like’ mostly inhabits slightly deeper Mwanza Gulf waters deprived of short-wavelength light with redshifted light conditions (Carleton et al., 2005; Meier et al., 2017; Wright et al., 2019). *H. sauvagei* is a depth-generalist species of Lake Victoria (Van der Meer, 1994; Seehausen, 1996; Gobbin et al., 2020). Visual properties such as the opsin genes' genetic sequences and the expression of genes involved in the visual system vary across these species (Wilwert et al., 2022; Wright et al., 2019), which could be driven by variation in visual habitats across species and may affect species-assortative mate choice (Seehausen et al., 2008). As the species inhabit different depth ranges, the visual environment differs between these species, especially with respect to the relative abundances of short-wavelength (blue) light (only present in the shallows) and long-wavelength (red) light (the dominant wavelength in deeper habitats). Therefore, here, we quantify the sensitivity to both blue and red lights.

Blue and red lights are particularly absorbed by the sws2ab (sws2a and sws2b) and lws opsins, respectively. Green light was used as a control light stimulus opposite the test light stimuli (blue and red lights) (as in Powers, 1978). Green light dominates the natural habitats of all three species and is particularly absorbed by the rh2a opsin, which is expressed on the retina of all three species included in this study (Spady et al., 2006; Wilwert et al., 2023). We did not use white light as a control light stimulus (as in some other studies that quantified relative sensitivity using the dorsal light response: Muntz et al., 1996; Silver, 1974) because white light contains a range of wavelengths, including the wavelengths of our test light stimuli, making the results difficult to interpret.

It must be noted that the assignment of blue and red lights as test light stimuli and green light as the control stimulus is an arbitrary choice, as the test and control lights are not treated differently during the experiment. Sensitivity to any colour of light is always measured relative to the sensitivity of another colour of light when using the dorsal light response. Therefore, a higher relative sensitivity to blue light, for example, can be because of a higher absolute sensitivity to blue light, a lower absolute sensitivity to green light, or both and is therefore equivalent to a lower relative sensitivity to green light. The green control light in this study is therefore not a ‘true’ control. For the sake of simplicity, we will refer to blue and red lights as test stimuli, always referring to the sensitivity of ‘test’ light relative to the green ‘control’ light.

### Fish housing, breeding and developmental light treatments

We used second- to fourth-generation male and female offspring from individuals collected in Lake Victoria (Wright et al., 2019) in 2014: at Python Island (2°37′26′′S, 32°51′24′′E; *Pundamilia* spp.) and Makobe Island (2°21′55′′S, 32°55′22′′E; *H. sauvagei*). Fish were kept in recirculation systems at 25±1°C in a 12L:12D light cycle and were fed twice a day (on the weekends once a day) with commercial cichlid pellets and frozen artemia. For details on breeding strategy, see Maan et al. (2017).

Fish were raised from ∼5 days after fertilisation in one of two developmental light treatments: a broad-spectrum light treatment, mimicking shallow-water ambient light spectra, and a redshifted light treatment, mimicking deep, turbid-water light spectra (Fig. 1A; Table S1; see Maan et al., 2017, for details on developmental light treatments). Fish were not bred specifically for this study but came from a pool used for multiple behavioural experiments. Individuals were raised until adulthood (i.e. ∼200 days of age) before relative sensitivity was tested. Age at the first trial ranged from 311 to 1768 days. Distribution of age was similar within species, for fish raised in different developmental light treatments, but differed slightly between species (Fig. S15). Age variation may introduce noise but was unavoidable due to limited availability of fish caused by fluctuations in breeding rates and logistical constraints. We accounted for this variation in the statistical analysis, as pedigree information was available for all fish (see below).

### Measuring the dorsal light response

We quantified the dorsoventral axis angle by exposing fish to different intensities of two light stimuli, one on each side of the fish (Fig. 2; Fig. S14). In total, 99 fish were tested (*n*=15–18 per species per developmental light treatment; Table S5). Each individual fish was tested three times on different days. To keep track of the individual fish, they were housed separately in their original developmental light treatment in between trials.

We used a panel with either blue (470 nm) or red (615 nm) light-emitting diodes (LEDs) as the test light stimuli, opposite a panel with green (525 nm) LEDs as the control light stimulus (Fig. 1B). LED panels were equipped with a coloured filter blocking the wavelength of the other light stimulus and a diffusion filter to diffuse the light of individual LEDs into a single uniform light field. Each fish was, within a single experimental trial, subjected to 15 different combinations of test light and control light intensities for each of the two test light colours (Fig. S16). Light intensities were adjusted by varying the voltage of the LED power supply to prevent the light stimuli from flickering at low intensities. Intensities of the combinations of test light and control light were, before the start of all trials, measured in the tube in which the fish was housed using a spectrometer (StellarNet BLACK Comet SR spectrometer, Tampa, FL, USA). The intensity of test and control light stimuli varied slightly depending on where in the tube of the experimental set-up, in which the fish was housed during the trial (Fig. 2A), was measured: the light intensity of test and control lights was slightly lower in the front of this tube than in the middle of the tube. However, the relative intensity of the test light over control light was stable over the tube (Fig. S16). We used relative light intensity measurements from the middle of the tube for the statistical analyses. The intensity of the test and control lights was around the same order of magnitude as light intensities in the native habitats of these fish, based on measurements in the Mwanza Gulf by Wright et al. (2019).

Each of the test and control intensity combinations for a specific colour (red or blue) was presented twice in each trial, within test light colours in a random order, until the fish was stable and positioned in such a way that the angle of the dorsoventral axis could be measured [i.e. facing a webcam (Logitech C925E, Lausanne, Switzerland) used to monitor the fish and record the trial, with one light stimulus illuminating each eye; stimuli were presented for at least 10 s]. After all combinations of light intensities for one of the test light colours were presented to the fish, the panel with test light LEDs was switched for the panel with the other test light colour. For 250 trials, the fish was presented first with the blue test stimuli; for the other trials (*n=*46), the red test stimuli were presented first. The position of the test and control lights was switched between trials of the same individual. After the trials, the dorsoventral axis angle (relative to the axis perpendicular to the water level) was measured for the different relative light intensities on screenshots of the recording of the trial using the Fiji software package (Schindelin et al., 2012). A tilt towards the test light was designated as a positive angle, regardless of whether the test light was to the left side or the right side of the fish. In some cases, a fish would not orient itself in such a way that the dorsoventral axis angle could be reliably measured. This occurred for one colour in five trials and for both colours in one trial. As a result, we had incomplete data for six fish (out of the 99 tested).

### Quantifying gene expression

We euthanised fish between 5 and 252 days (with a median of 30 days) after the final experimental trial using a lethal dose of tricaine methanesulfonate (MS-222; 1 g/l, dissolved in water from the recirculation system; Sigma-Aldrich Co., St. Louis, MO, USA) buffered with sodium bicarbonate (1 g/l) and subsequent cutting of the spinal column. A minimum sampling delay of 5 days was used to minimise the effect of any short-term effects of the behavioural trials on visual gene expression patterns.

Eyes were dissected and preserved separately in either RNAlater (Invitrogen, Waltham, MA, USA) or NucleoProtect RNA (BIOKÉ, Leiden, The Netherlands), stored overnight at 4°C and subsequently frozen at −20°C until further processing. Euthanasia and eye dissection were carried out in the same light conditions as in the developmental light treatment. To control for circadian rhythms in opsin expression levels (Halstenberg et al., 2005; Yourick et al., 2019) and maximise RNA yield, we euthanised all fish between 15:00 and 18:00 CET. One individual died before it could be sampled.

Eyes were thawed, and the retina was removed for total RNA isolation using TRIzol (Invitrogen) and chloroform. The final RNA concentration was measured on a NanoDrop One microvolume UV-Vis spectrophotometer (Thermo Fisher Scientific, Waltham, MA, USA). Subsequently, we reverse transcribed 1 μg of extracted RNA into cDNA using Oligo(dT)18 primers (Thermo Fisher Scientific) and RevertAid H Minus Reverse Transcriptase (Thermo Fisher Scientific) at 45°C. The resulting cDNA was diluted to a final concentration of 10 ng/μl.

Using quantitative real-time PCR (qPCR), we quantified the expression of four different cone opsin genes encoding different opsin proteins, which absorb different wavelengths of light (Carleton et al., 2016): the short-wavelength-sensitive opsin 2b (*sws2b*, violet light) and 2a (*sws2a*, blue light), rhodopsin-like 2a (*rh2a*, green light, combining the expression of the genetically and functionally similar *rh2aα* and *rh2aβ*, see Hofmann et al., 2010; O'Quin et al., 2010; Spady et al., 2006; Wilwert et al., 2022, 2023; Wright et al., 2019; Wright et al., 2020) and the long-wavelength-sensitive opsin (*lws*, orange-red light). These four genes dominate cone opsin expression in the three species used here; haplochromine cichlids may express two other cone opsin genes, but these are negligible in our study species (generally <0.5% of total cone opsin expression; see O'Quin et al., 2010).

We also quantified the expression of the cytochrome P450 family member *cyp27c1* as a proxy for the ratio of vitamin-A_1_- to vitamin-A_2_-derived chromophore (Enright et al., 2015; Torres-Dowdall et al., 2017). As reference genes, we quantified the expression of L-lactate dehydrogenase 1 (*ldh1*), glyceraldehyde-3-phosphate dehydrogenase 2 (*gapdh2*) and *β-actin* (Jin et al., 2013; Torres-Dowdall et al., 2017; Wilwert et al., 2022, 2023).

After a denaturation step (95°C for 3 min), gene-specific qPCRs, with 1 μl of cDNA (10 ng/μl) added to the reaction mix, were run for 50 cycles (denaturation at 95°C for 15 s, primer annealing at 60°C for 25 s, extension at 72°C for 30 s). qPCR was followed by a dissociation curve analysis. Fluorescence was monitored on a StepOnePlus Real-Time PCR System (Applied Biosystems, Waltham, MA, USA) using SYBR Green as a dsDNA binding dye (iQ SYBR Green Supermix, Bio-Rad Laboratories, Hercules, CA, USA). All samples were run in duplicate. See Table S6 for all primer sequences.

We estimated the initial concentration of cone opsin, *cyp27c1* and reference gene transcripts in the retina using the ‘window-of-linearity’ method (Ramakers et al., 2003; Ruijter et al., 2009). For quality control criteria, we only included samples where the standard deviation in the *C_q_* of the duplicates was less than 0.5, and where the individual PCR efficiency deviated by less than 15% from the mean overall PCR efficiency (Wilwert et al., 2022, 2023).

The peaks of the spectra of blue and red test lights used in the experimental trials were close to the peak of the opsin absorption spectra of the sws2ab (sws2a and sws2b) and lws opsins, respectively. The spectrum of the green control light is close to the peak of the absorption spectrum of the rh2a pigment (Fig. 1). The expression ratios of *sws2ab* (*sws2a* and *sws2b* combined) to *rh2a* and *lws* to *rh2a* are therefore hypothesised to align with the relative sensitivity to the blue and red test lights, respectively. Therefore, we calculated the expression ratios of *sws2ab* to *rh2a* and *lws* to *rh2a* and transformed these ratios with the natural logarithm. For cyp27c1, this logic does not apply: the expression of *cyp27c1* was therefore normalised against the geometric mean of the three reference genes’ expression. Opsin expression ratios and normalised *cyp27c1* expression were visually inspected for outliers, and no outliers were detected. The sample sizes for *cyp27c1* expression were slightly lower than the opsin expression ratio due to the quality control criteria; however, the missing data for *cyp27c1* did not introduce biases in sample size across groups (Table S7).

### Statistical modelling

We analysed the dorsal light response using linear mixed-effects models, in which the dorsoventral axis angles of the different species and fish raised in different developmental light treatments were predicted as a function of relative light intensity. We estimated the tipping point (i.e. the relative light intensity where the dorsoventral axis angle equals 0°) from the slope and intercept for the response of the dorsoventral axis angle to relative light intensity. We assessed differences in relative light sensitivity by comparing this tipping point among groups (Muntz et al., 1996; Powers, 1978; Silver, 1974).

We assessed variation in the dorsal light response using linear mixed-effects models. Dorsoventral axis angle was used as the dependent variable, with the relative light intensity of the stimulus lights (transformed with the natural logarithm), species and developmental light treatment, and the two-way and three-way interaction terms between these factors as independent factors. We included an interaction between species and age of the fish at the time of the behavioural trial by species. Additionally, the absolute intensity of the control light was incorporated into the models to determine whether absolute light intensity affects the angle of the dorsoventral axis. Separate models were fitted to account for relative sensitivity to blue and red light.

To test whether relative sensitivity is correlated with gene expression, we added opsin expression ratios (*sws2ab* over *rh2a* and *lws* over *rh2a* for blue- and red-light sensitivity, respectively) and *cyp27c1* expression level (for both blue- and red-light sensitivity), as well as the interaction between them, as fixed effects to the models. Even though opsin expression patterns are fairly stable over time in adult fish (Irazábal-González et al., 2024; Wilwert et al., 2023), we controlled for the effects of elapsed time between the last behavioural trial and eye sampling by including this delay in the statistical model. This sampling delay was included in the model both as an interaction with gene expression patterns and in a three-way interaction between the opsin expression ratio and the relative light intensity.

During the experiments, we observed differences in the strength of the dorsal light response across individuals and across trials (see also Pfeiffer and Riegelbauer, 1978; Von Holst, 1948). To control for these differences, the identifiers of both the individual fish and the trials were included as random intercepts. Additionally, the effect of relative light intensity on angle was allowed to vary across individuals and trials (i.e. a random slope for relative light intensity was fitted for each fish and trial; Schielzeth and Forstmeier, 2009). To account for relatedness among individuals, the identifiers of the clutches of the fish were included in the models as random intercepts. Additionally, the position of the test light during the trial (on the left or right side of the fish, as lateralisation may result in differences between the left and right eyes; Miletto Petrazzini et al., 2020) and the sex of the fish were fitted as random intercepts. We chose to add sex as a random effect instead of a fixed effect because the number of males and females was not evenly distributed over the experimental groups due to the limited availability of fish (Table S5). We therefore lacked the statistical power to assess sex-related differences in relative light sensitivity. Age of the fish was fitted as a random slope, separately for the three species. Finally, to account for any changes in dorsal light responses across replicate behavioural trials, the number of replicates in each trial was modelled as a random slope for each species. For random-effect predictions of all models, see Tables S8, S9 and S10. Post hoc tests on fixed effects were performed using Tukey's honestly significant difference tests. Separate models were fitted for relative sensitivity to blue- and red-test-light stimuli.

The tipping points of different groups were calculated based on the predictions of slopes and intercepts of the response of the dorsoventral axis to relative light intensity of the statistical models, which were obtained from the fixed-effect estimates in the models. Standard errors of tipping points were calculated using the delta method (Dorfman, 1938) using the covariance matrix of the statistical model. For the models testing the relation between visual sensitivity and visual gene expression, the tipping points were calculated as a function of gene expression patterns. Standard errors of tipping points were used to test whether the tipping points differed significantly between experimental groups; *P*-values for these tests were corrected for multiple comparisons using a Holm (1979) correction. To test whether tipping points changed as a function of gene expression patterns, we tested whether the tipping point differed between fish with the lowest and fish with the highest opsin expression ratio/*cyp27c1* expression, again based on the model predictions of slopes and intercepts and using the delta method to calculate standard errors of tipping points. For visualisation purposes, we estimated confidence intervals of the tipping points by bootstrapping the models (10,000 replications).

All statistical analyses were performed using R version 4.4.1 (R Core Team, 2023). Linear mixed-effects models were fitted using *lme4* v1.1-35.5 (Bates et al., 2015). The package *car* (Fox and Weisberg, 2019) was used for the delta method to calculate standard errors of the tipping points. Post hoc tests in expression levels were performed using *emmeans* v1.10.5 (Lenth, 2023). Model bootstrapping was performed using the package *boot* v1.3-30 (Canty and Ripley, 2022; Davison and Hinkley, 1997).

## Supplementary Material



10.1242/biolopen.062674_sup1 Supplementary information
