# Supplementary material for: Correlating relative light sensitivity to retinal visual gene expression patterns in three African cichlid fish species
Source: Biol Open. 2026 Oct 12;15(10):bio062674. doi: 10.1242/bio.062674 (PMC13714874; doi:10.1242/bio.062674)
Supplement: Supplementary file 1 [file biolopen-15-062674-s1.pdf]

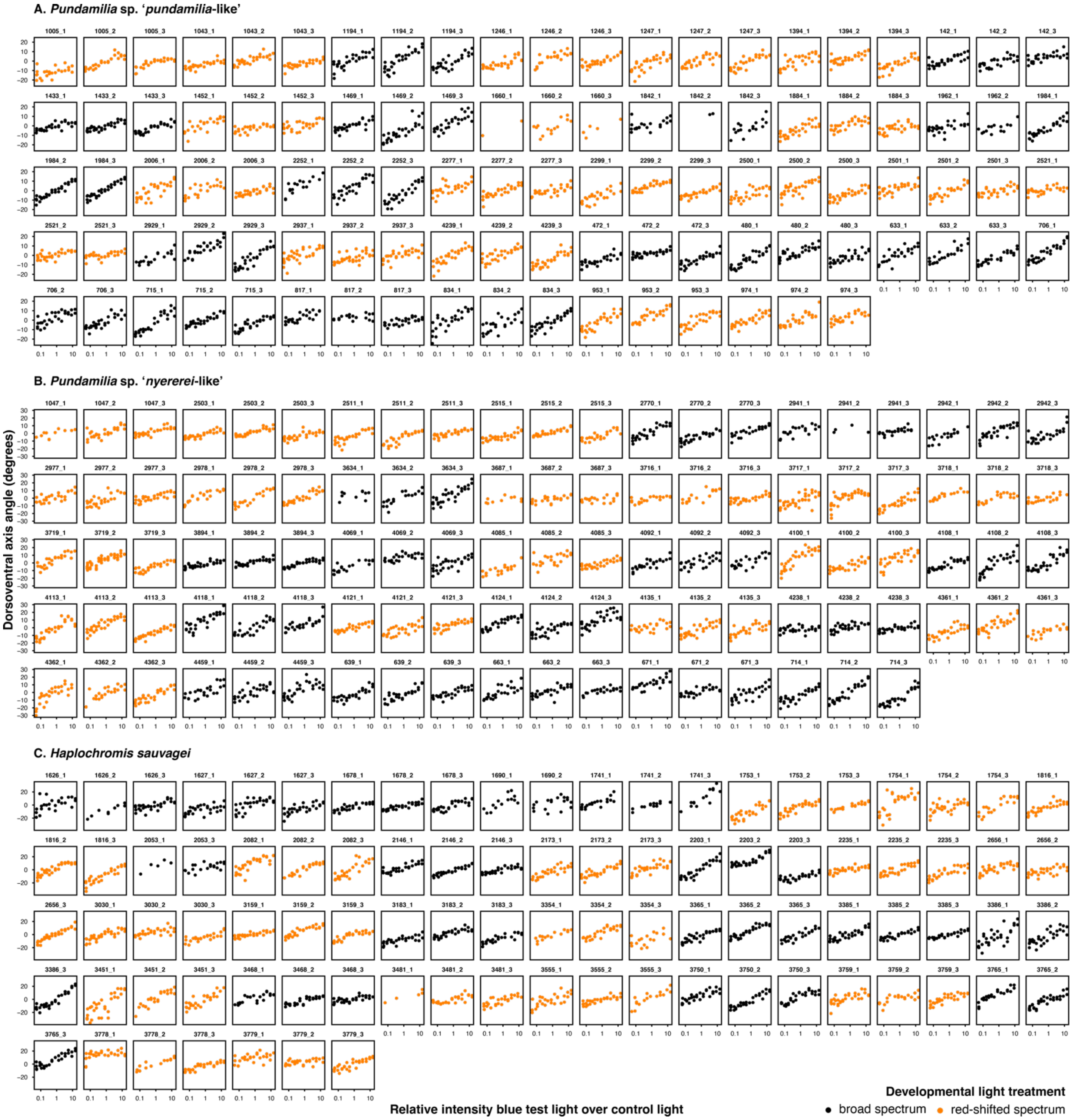

Fig. S1. Dorsoventral axis angle response to relative light intensity for the blue test light stimulus for each behavioural trial separately. Numbers above the plots refer to individual identifiers of the fish, followed by the trial number for that fish. Trials of the same fish are plotted next to each other.

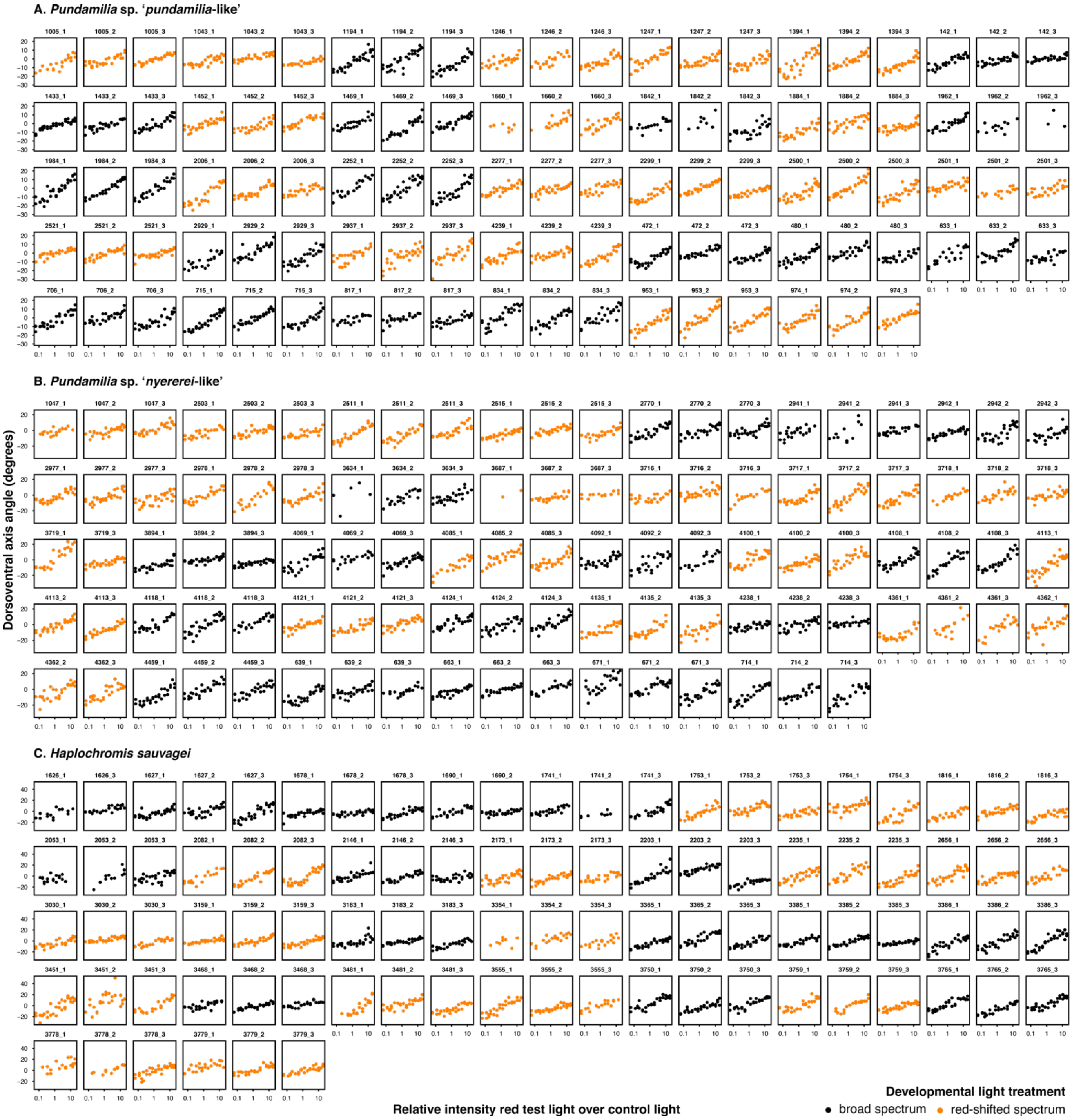

Fig. S2. Dorsoventral axis angle response to relative light intensity for the red test light stimulus for each behavioural trial separately. Numbers above the plots refer to individual identifiers of the fish, followed by the trial number for that fish. Trials of the same fish are plotted next to each other.

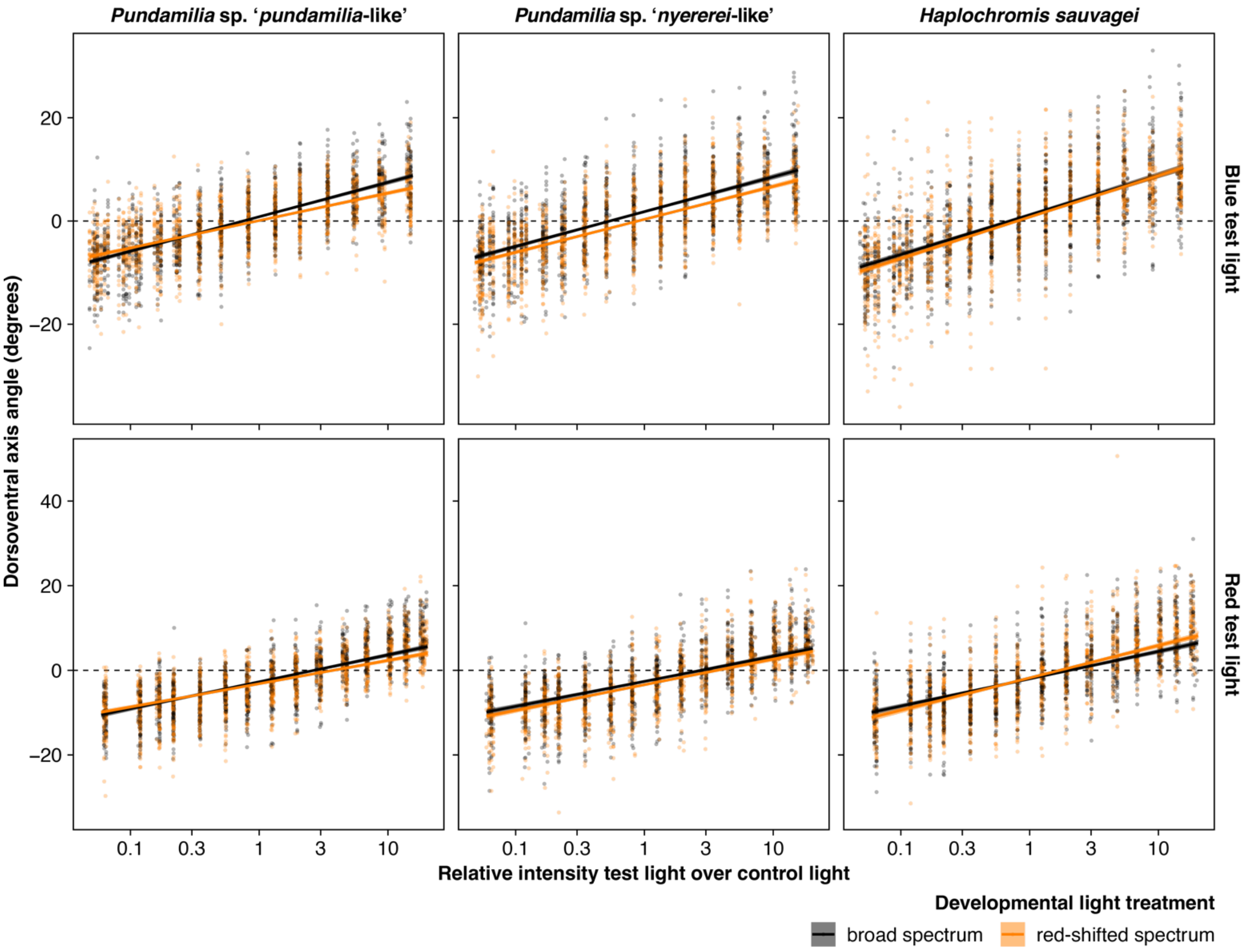

Fig. S3. Dorsoventral axis angle across relative intensities of blue (top panels) and red (bottom panels) light stimuli in fish raised in different developmental light treatments. Confidence bands are based on bootstrapping a full model (n = 10,000; bands contain 95% of bootstrapped estimates).

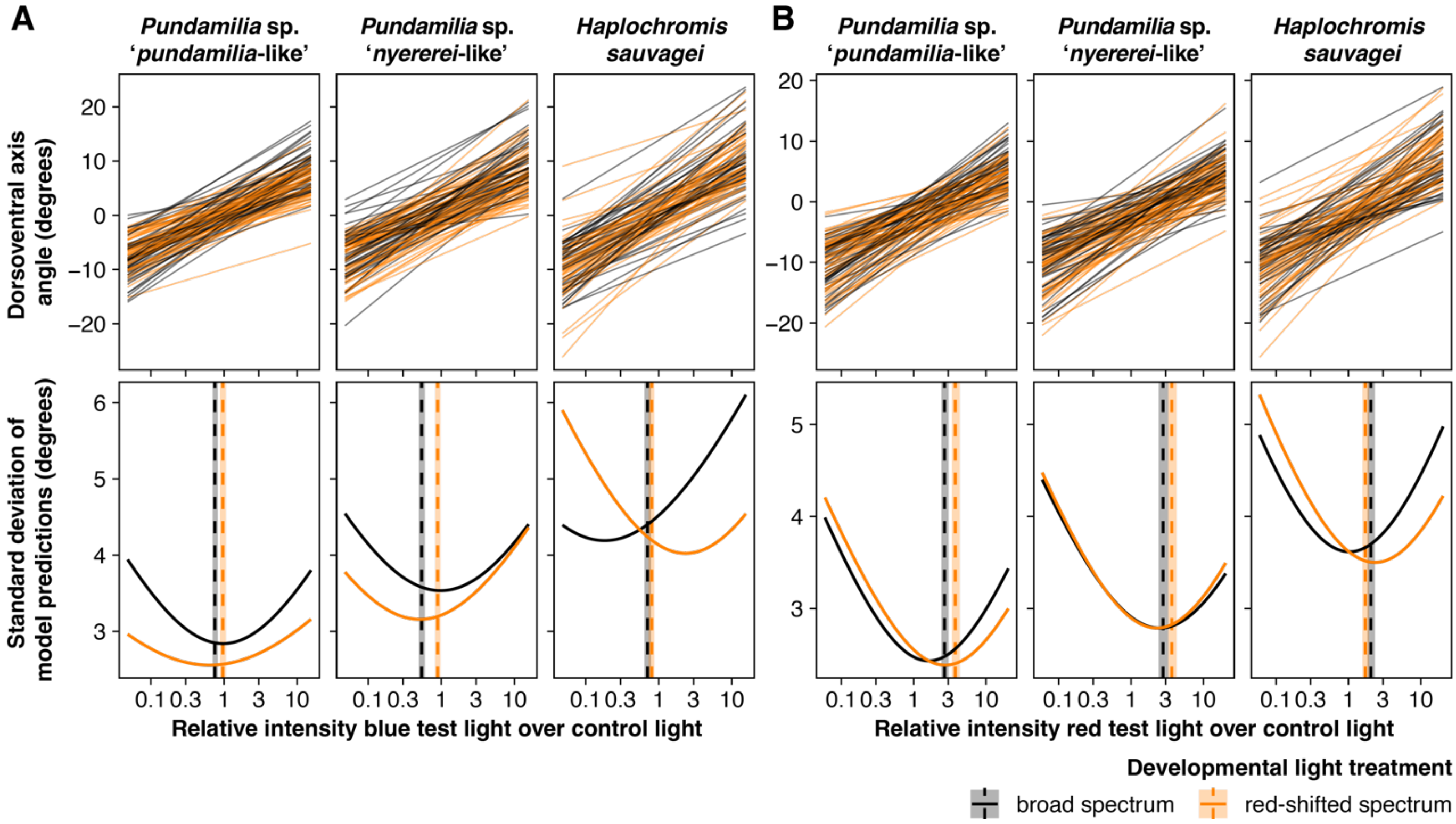

Fig. S4. Model predictions of the dorsoventral axis angle response to relative light intensity for each behavioural trials, based on the random effects in the statistical model (top row), and the standard deviation of these predictions (bottom row) for the blue test light (A) and the red test light (B). In the bottom row, the position of the group-level estimations of the tipping points are shown with vertical dashed lines, the confidence bands around this lines are based on bootstrapping (n = 10,000; shaded areas contain 95% of bootstrapped estimates, see also Fig. 5).

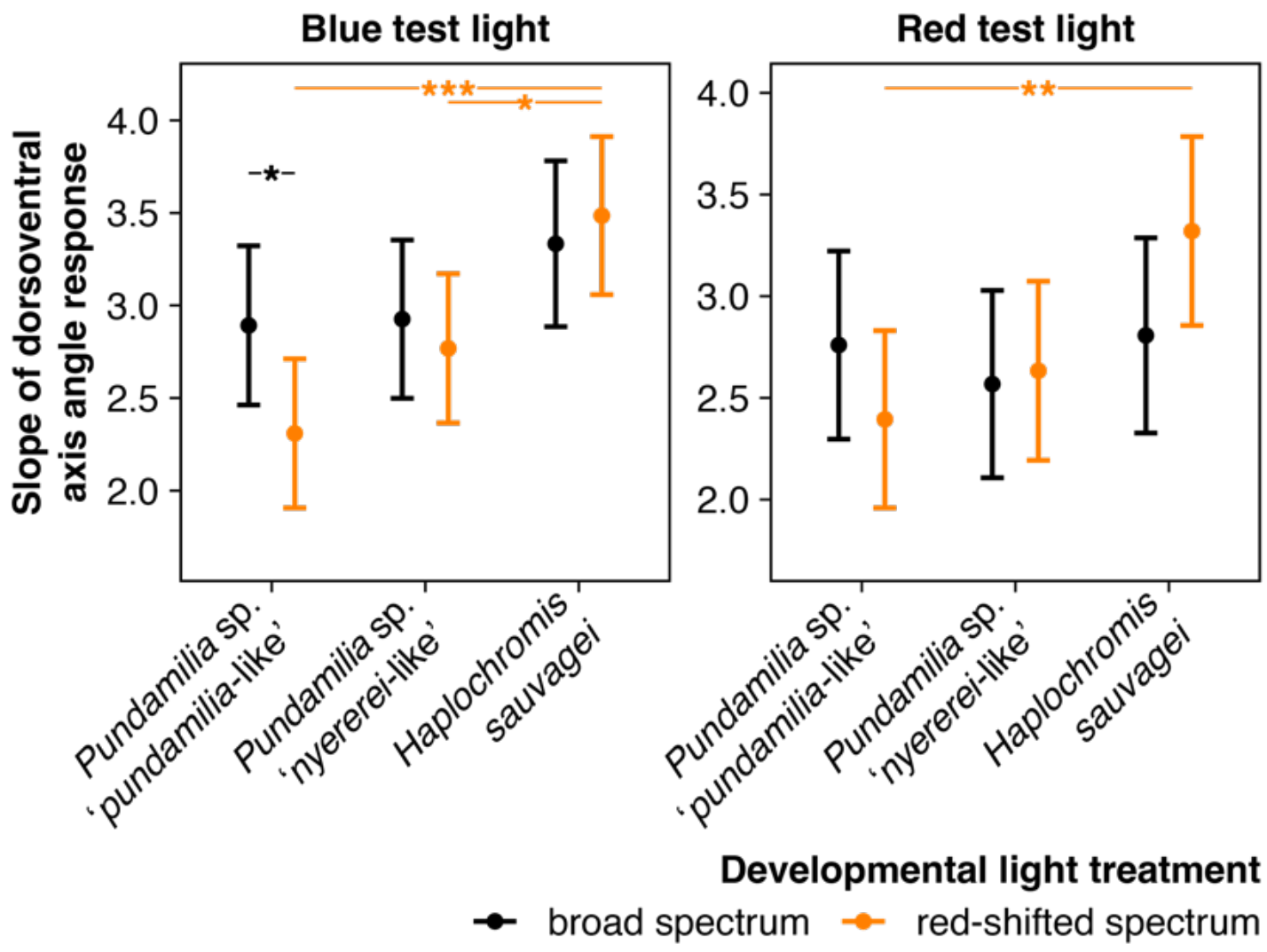

Fig. S5. Predicted developmental light effect and the species-effect on the slope of the dorsoventral axis angle response to blue and red test light. Effects are estimated for fish with a median age, and for median control light intensities. Errorbars are based on bootstrapping (n = 10,000; errorbars contain 95% of bootstrapped estimates). Contrasts between groups: *: $p < 0.05$; **: $p < 0.01$; ***: $p < 0.001$.

Biology Open • Supplementary information

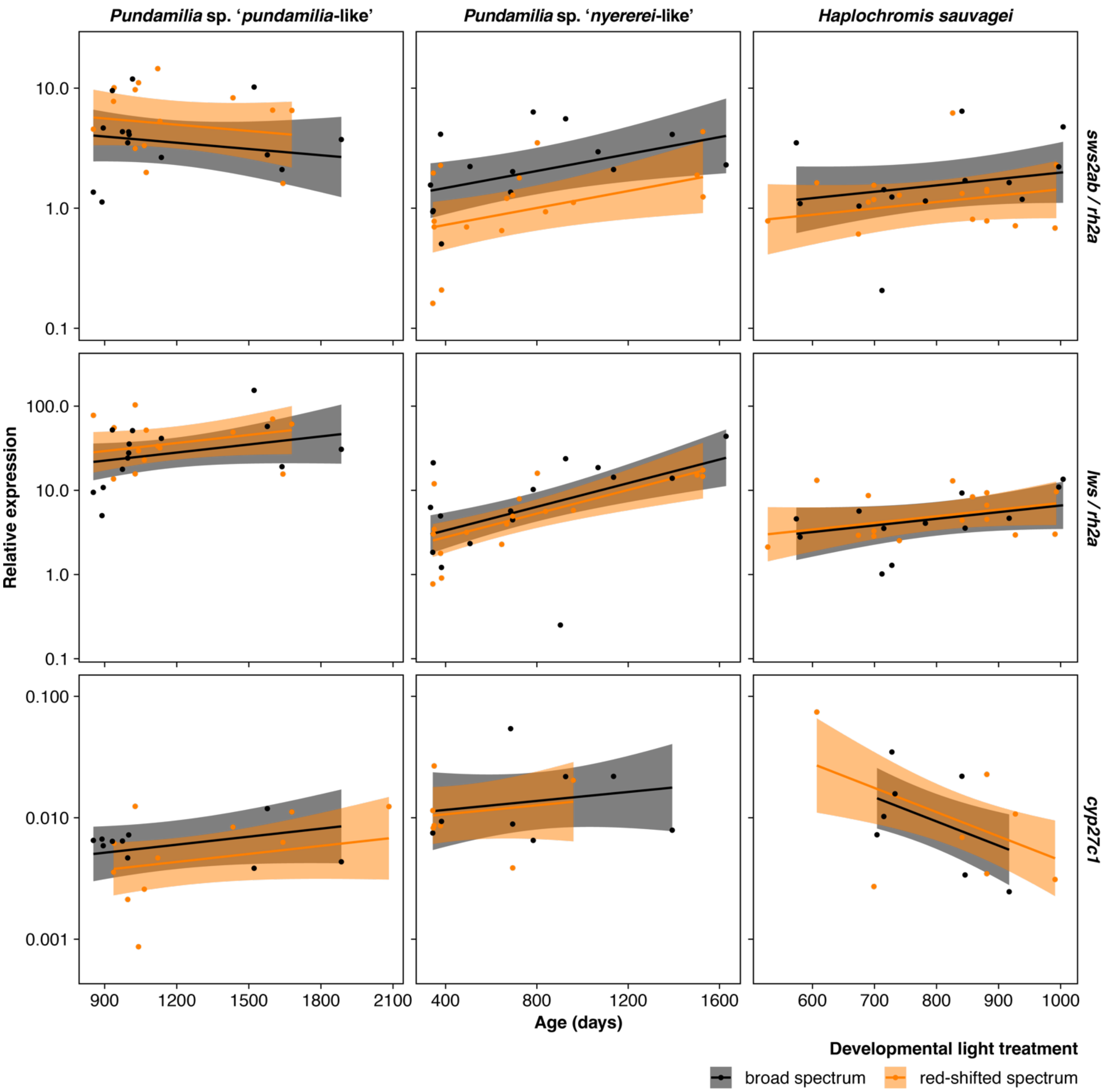

Fig. S6. Effect of age on opsin expression ratio and *cyp27c1* expression in fish raised in different developmental light treatments. Confidence bands denote two standard errors (estimated for a median age and delay between behavioural trials and retinal sampling), symbols denote individual observations.

Biology Open • Supplementary information

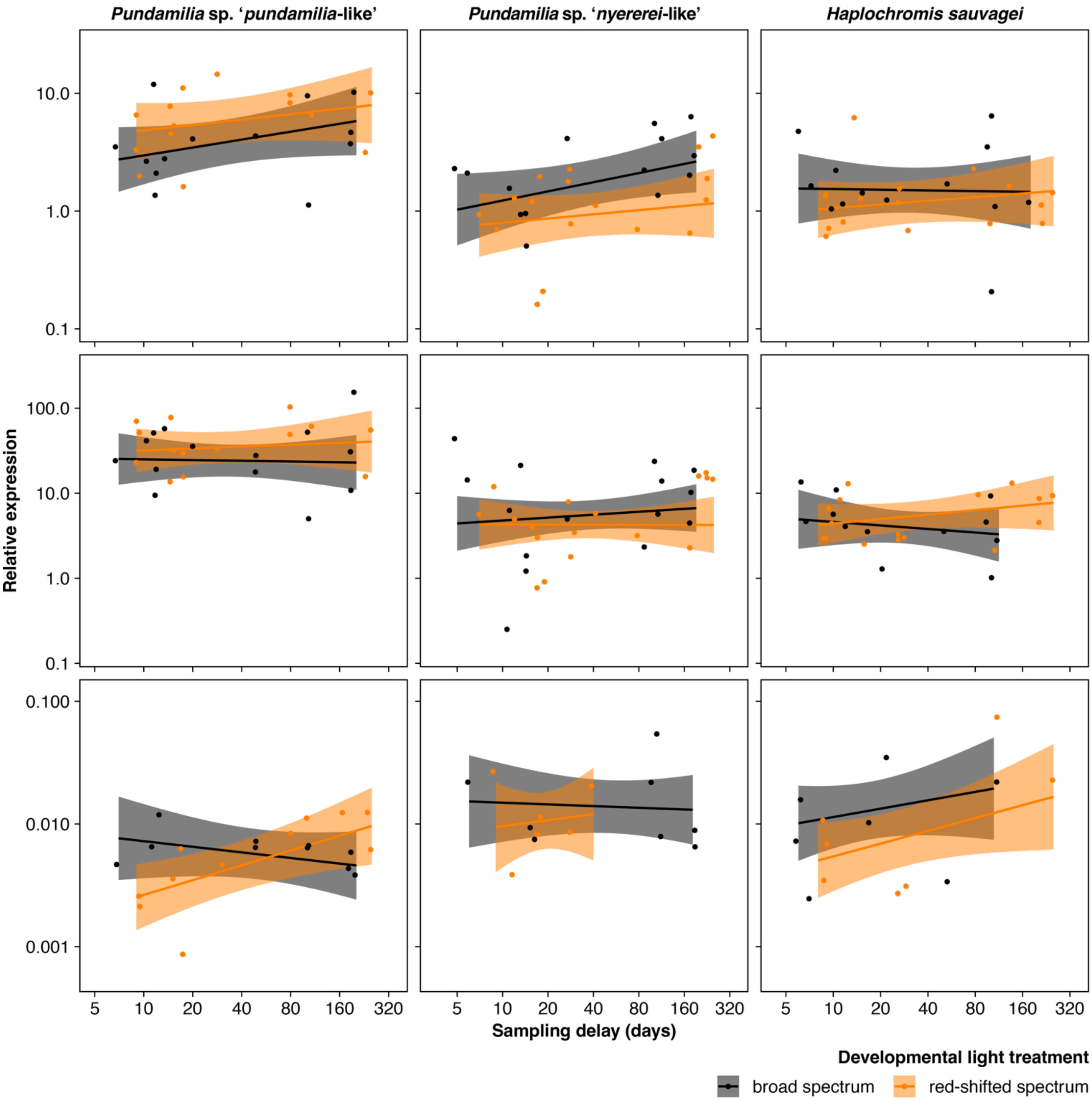

Fig. S7. Effect of sampling delay on opsin expression ratio and *cyp27c1* expression in fish raised in different developmental light treatments. Confidence bands denote two standard errors (estimated for a median age and delay between behavioural trials and retinal sampling), symbols denote individual observations.

Biology Open • Supplementary information

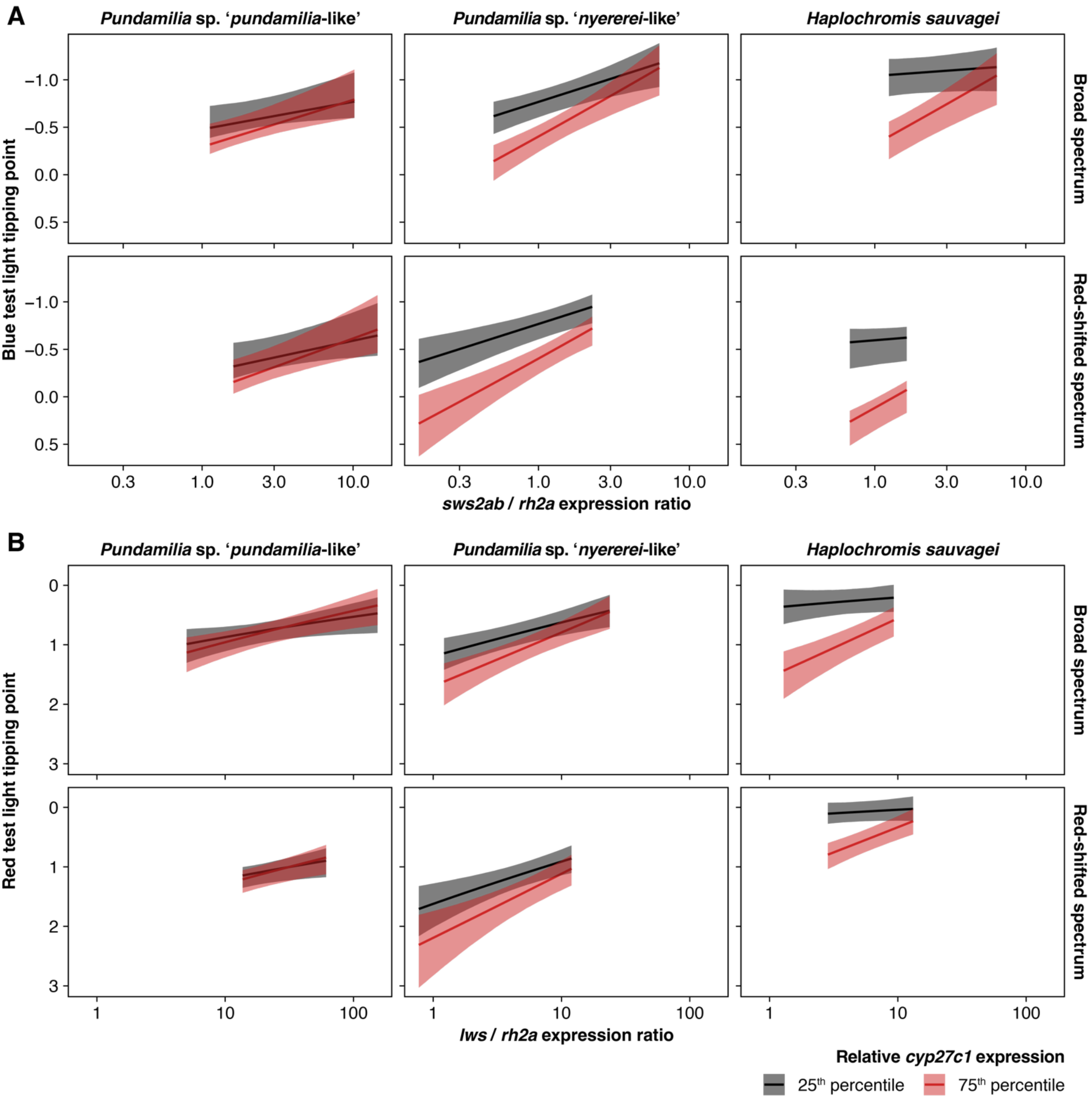

Fig. S8. Estimated relation between the tipping point for blue and red test light and opsin gene expression ratios. Predictions are shown for different levels of *cyp27c1* expression. The relation is estimated for a sampling delay of 7 days and for a median control light intensity and age. Confidence bands are based on bootstrapping estimates (n = 10,000; bands contain 95% of bootstrapped estimates). Note that the tipping point axis is reversed, with lower values corresponding to higher relative light intensities.

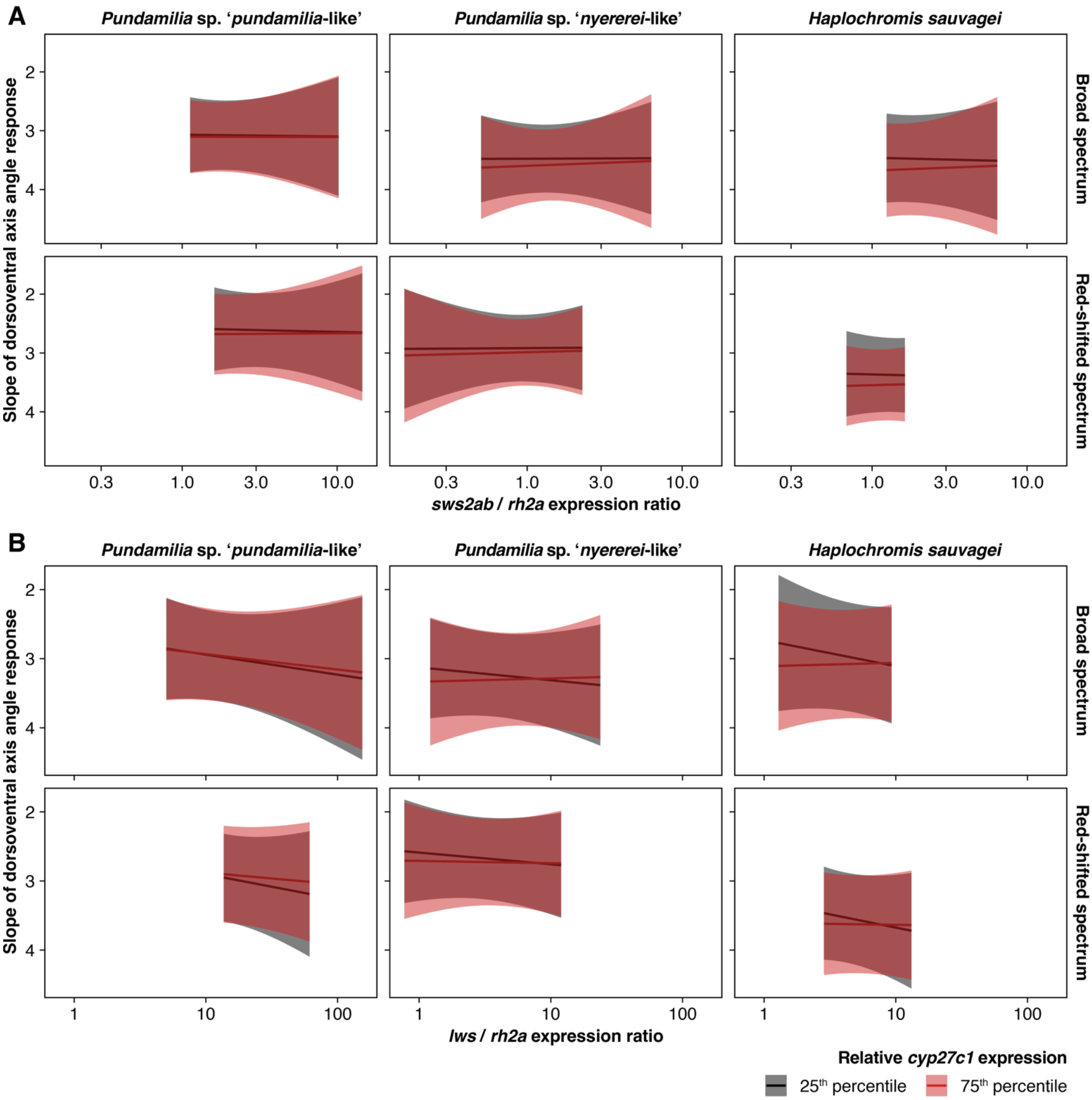

Fig. S9. Estimated relation between the slope of the dorsoventral axis angle response to blue (A) and red (B) test light and opsin gene expression ratios. Predictions are shown for different levels of *cyp27c1* expression. The relation is estimated for a sampling delay of 7 days and for a median control light intensity and age. Confidence bands are based on bootstrapping estimates (n = 10,000; bands contain 95% of bootstrapped estimates). Note that the tipping point axis is reversed, with lower values corresponding to higher relative light intensities.

Biology Open • Supplementary information

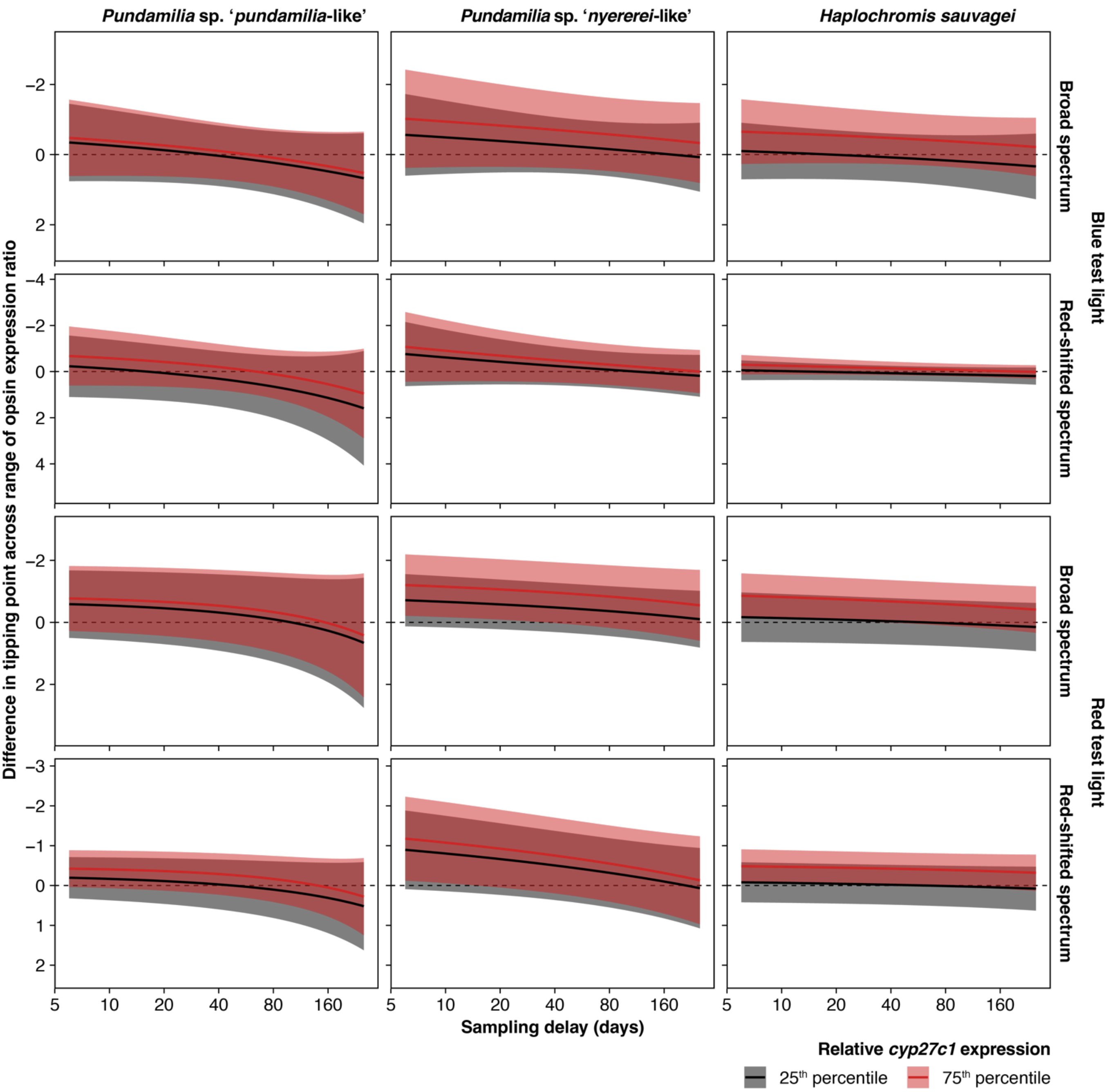

Fig. S10. Effect of sampling delay on the effect of opsin expression ration on the dorsoventral axis angle, based on model predictions, for both the blue and red test light (effect of *sws2ab* / *rh2a* and *lws* / *rh2a* expression ratio respectively). Predictions are shown for different levels of *cyp27c1* expression, and for a median control light intensity and age. In general, effect of the opsin gene expression ratio on the dorsal light effect becomes weaker with an increasing sampling delay. Shaded bands denote 95% confidence intervals.

Biology Open • Supplementary information

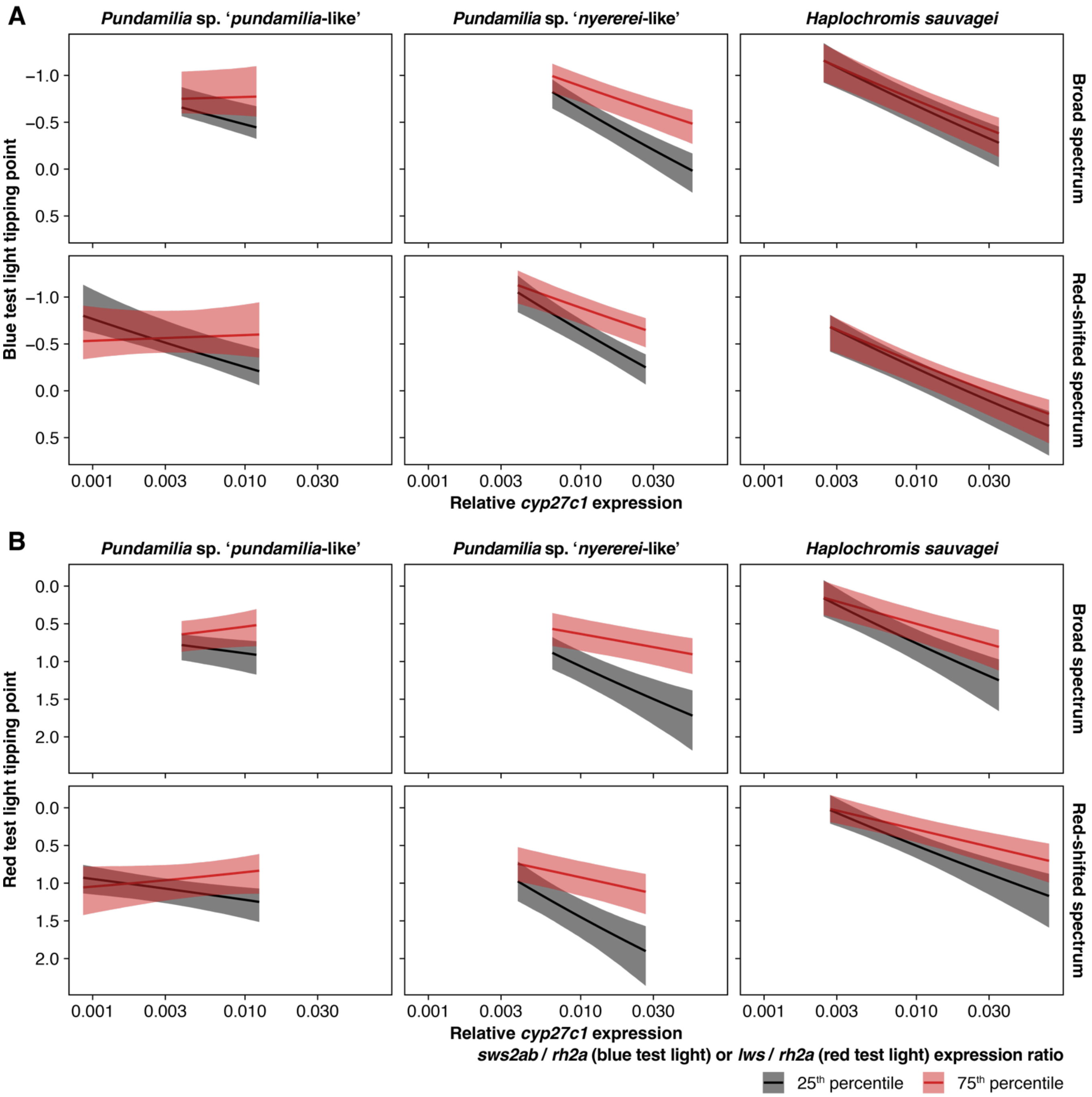

Fig. S11. Estimated relation between the tipping point for blue (A) and red (B) test light and relative *cyp27c1* expression. Predictions are shown for different opsin expression ratios. The relation is estimated for a sampling delay of 7 days and for a median control light intensity and age. Confidence bands are based on bootstrapping estimates (n = 10,000; bands contain 95% of bootstrapped estimates). Note that the tipping point axis is reversed, with lower values corresponding to higher relative light intensities.

Biology Open • Supplementary information

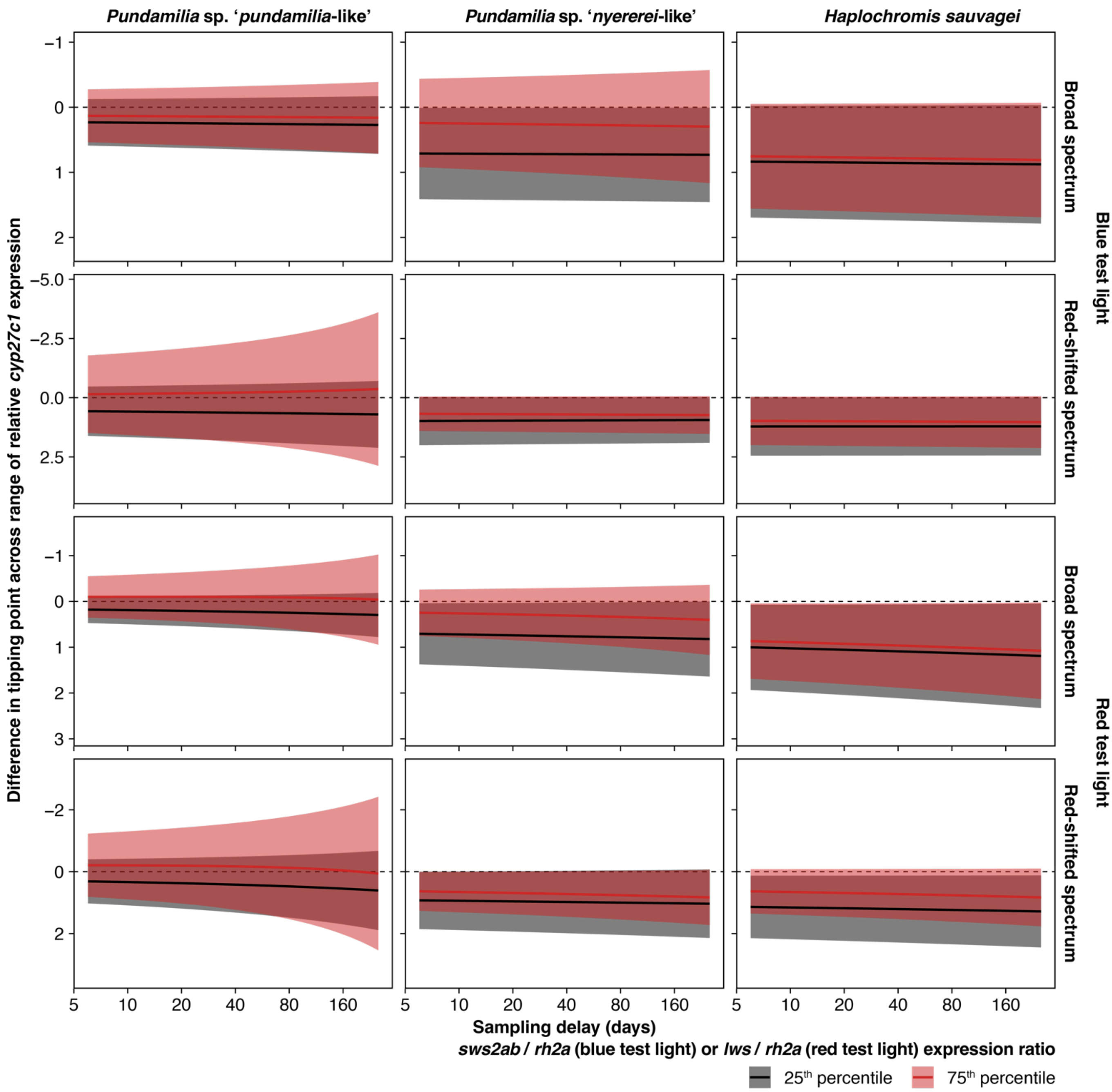

Fig. S12. Effect of sampling delay on the effect of relative *cyp27c1* on the dorsoventral axis angle, based on model predictions, for both the blue and red test light. Predictions are shown for different ratios of opsin expression levels, and for a median control light intensity and age. Shaded bands denote 95% confidence intervals.

Biology Open • Supplementary information

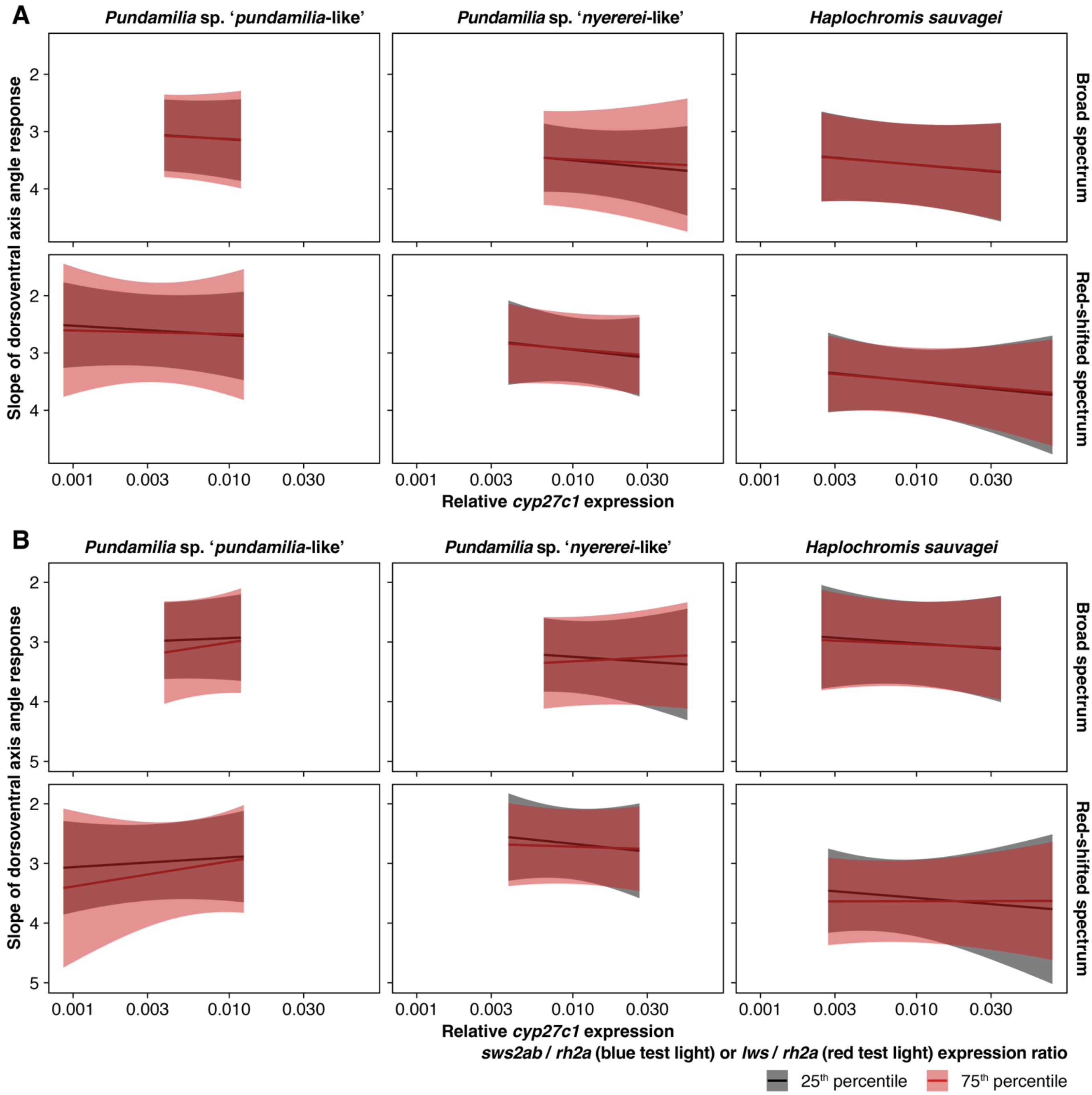

Fig. S13. Estimated relation between the slope of the dorsoventral axis angle response to blue (A) and red (B) test light and relative *cyp27c1* expression. Predictions are shown for different opsin expression ratios. The relation is estimated for a sampling delay of 7 days and for a median control light intensity and age. Confidence bands are based on bootstrapping estimates (n = 10,000; bands contain 95% of bootstrapped estimates). Note that the tipping point axis is reversed, with lower values corresponding to higher relative light intensities.

Biology Open • Supplementary information

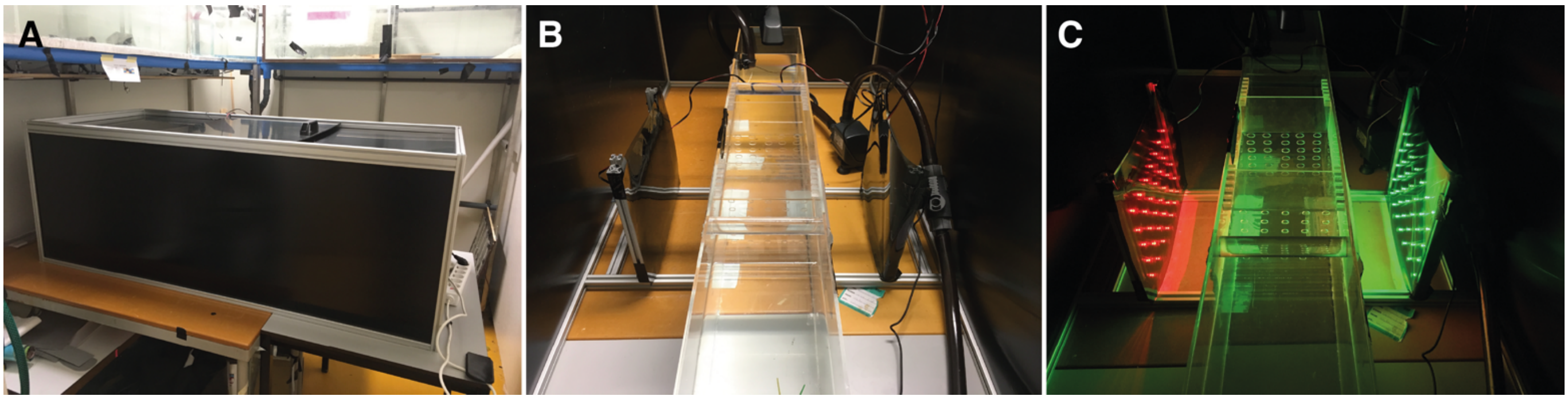

Fig. S14. Experimental setup to quantify relative behavioural sensitivity to different light stimuli using a fish' dorsal light response. (A): experimental box around the tank (1440 × 690 × 650 mm), to prevent external light from affecting the experimental trial; (B): experimental tank (1058 × 158 × 254 mm, constructed from 4 mm UV-transmitting acrylic) in with the LED panes (left and right) and the pump (right; Eheim compactON 3000, Deizisau, Germany) to generate water flow; (C): red light stimulus (left) and the green control stimulus (right). Some details were changed in the actual experimental trials which are not shown on the picture: i): the dividers between the tank compartments were changed and a tube was installed in the middle compartment to hold the fish (235 mm long, with a diameter of either 40 mm, 60 mm or 80 mm, depending n the size of the fish); ii): the bottom of the box was covered with black PVC, iii): coloured filters (see Table S2) and a diffusion filter were installed between the LED panels and the tank to prevent internal reflections and diffuse light from the stimuli respectively; iv): the neutral density filters that can be seen on the LED panels in photos b and c were removed in the final setup; v): the space between the light stimuli and the tank was encased with black PVC and black cloth to direct the light more efficiently towards the fish and prevent reflections.

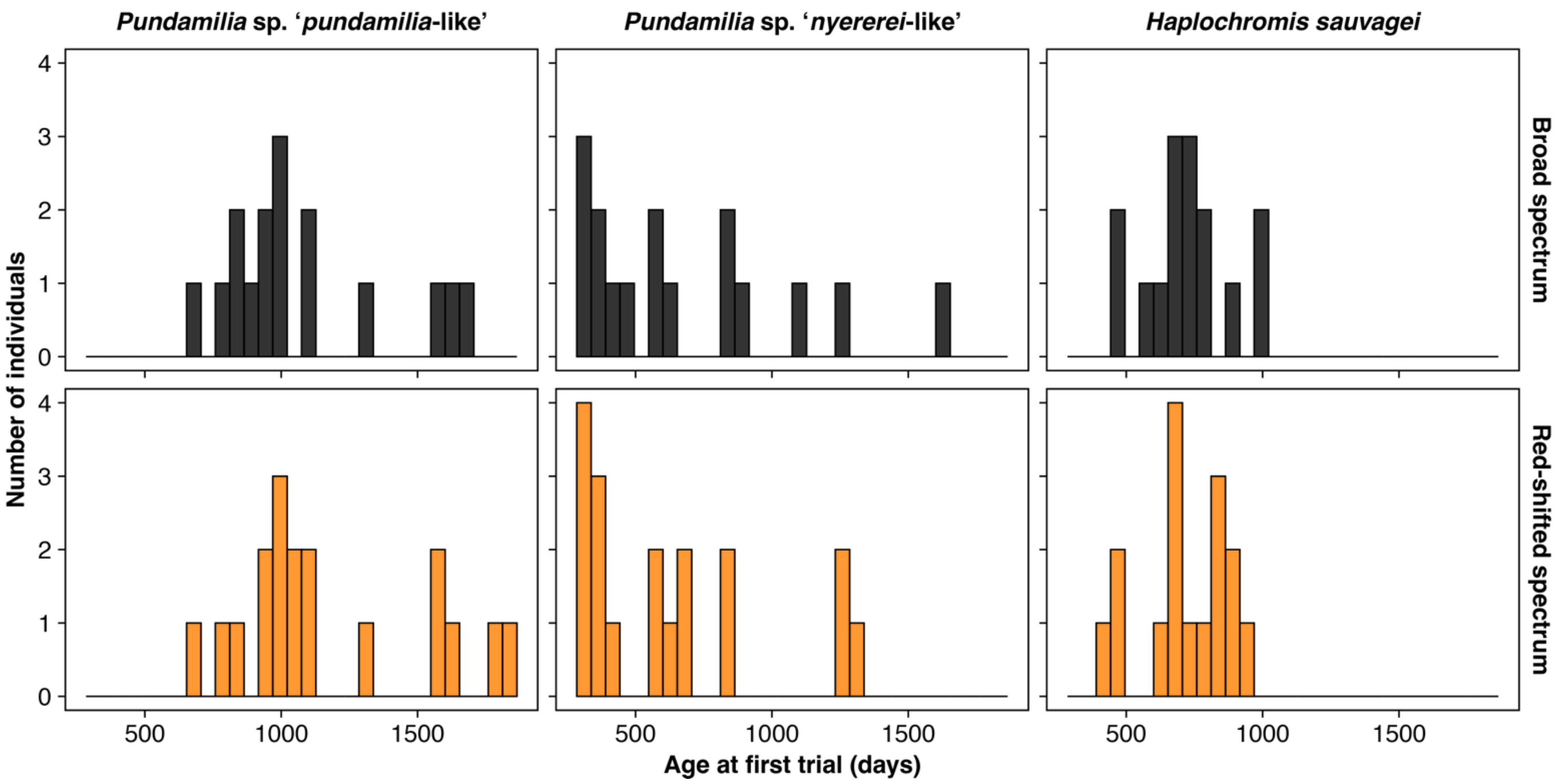

Fig. S15. Distribution of age at the first trial for each experimental group.

Biology Open • Supplementary information

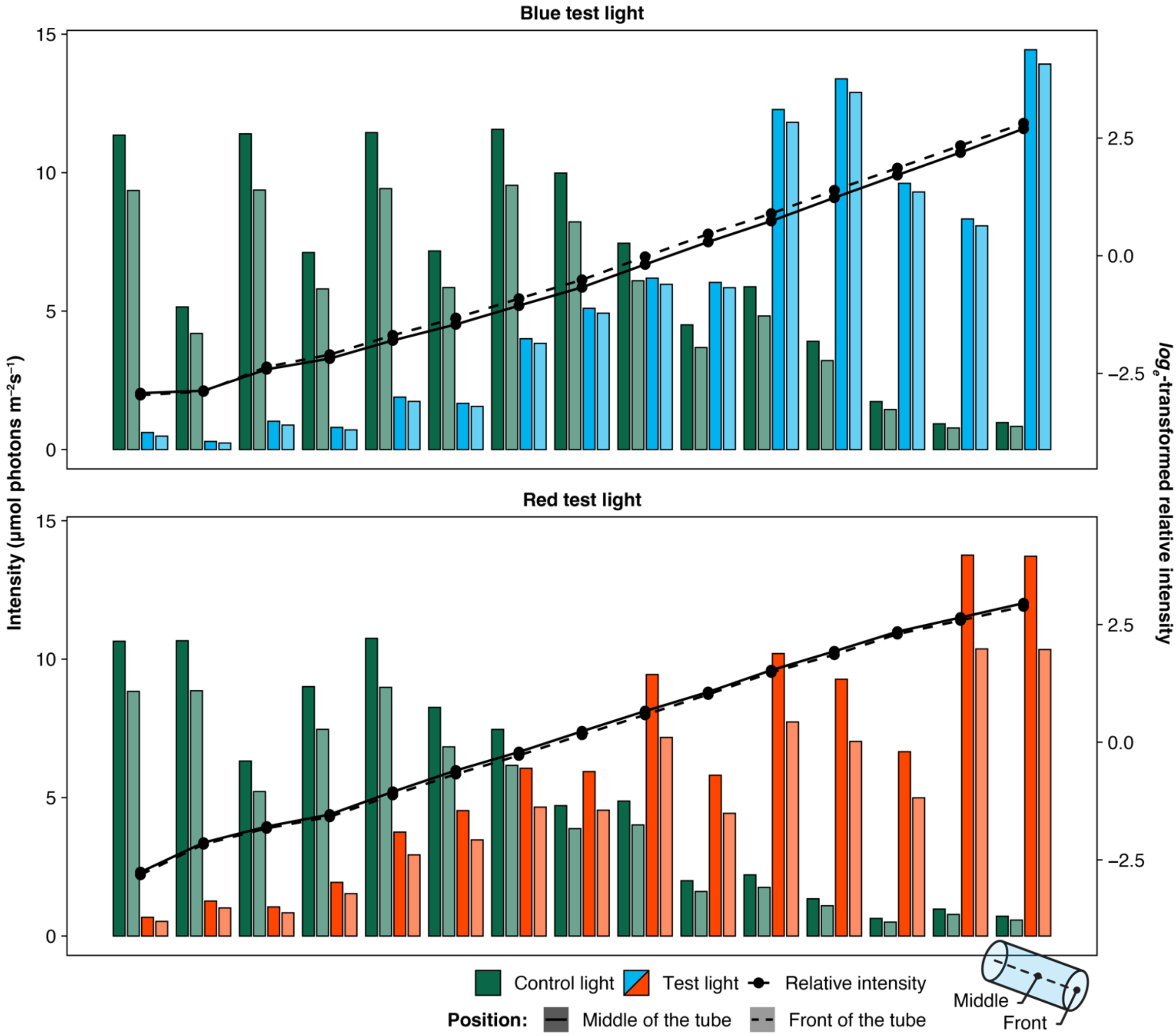

Fig. S16. Intensity of the control and test light (bars; left axis) and the relative intensity of test over control light (black symbols and line; right axis; transformed with the natural logarithm) in the 15 intensities presented to the fish. Intensities are shown for two positions in the tube in which the fish is housed during the trials: in the middle of that tube (dark colours, solid line), and at the front of the tube (lighter colours, dashed line; see Fig. 2). Intensities are averaged over the different layouts of the experimental setup (differently sized tubes to hold the fish, test light on the left or right side of the fish). For the analysis of spectral sensitivity, the non-averaged values were used, based on the experimental settings used for each individual fish.

Biology Open • Supplementary information

Table S1. Lights and coloured filters used to generate light stimuli and the developmental light treatments. All filters are manufactured by LEE (Andover, UK). See also Fig. 1. For details on light treatments, see also Maan (2017).

Light	Light source	Colour filter
Blue test light	Broadcom HLMP-CB2A-VW0DD (470 nm test light) opposite Broadcom HLMP-CM3A-Z10DD (525 nm control light)	LEE #716 (test-light)<br>LEE #767 (control light)
Red test light	Broadcom HLMP-EH3A-WX0DD (615 nm test light) opposite Broadcom HLMP-CM3A-Z10DD (525 nm control light)	LEE #019 (test light)<br>LEE #124 (control light)
Broad spectrum	Philips Masterline ES, 30 W and 35 W, or Osram DecoStar Titan 51 35W + Paulmann 88090 ESL Blue Spiral 15 W or Philips 929001394158 3.1W[*]	LEE #243
Red-shifted spectrum	Philips Masterline ES, 30 W and 35 W, or Osram DecoStar Titan 51 35W	LEE #243 and #015

[*]: blue lights were added to the broad spectrum condition to compensate for the lack of short-wavelength light in the halogen lights

Table S2. Results of post-hoc tests on the effect of species and developmental light treatment on the intercept, slope and tipping point of the dorsoventral axis response to blue and red light. Significant differences ($p < 0.05$) are marked in bold.

		Blue test light						Red test light					
		Intercept		Slope		Tipping point		Intercept		Slope		Tipping point	
**Contrast**	**Group[*]**	***z*-ratio**	***p***	***z*-ratio**	***p***	***z*-ratio**	***p***	***z*-ratio**	***p***	***z*-ratio**	***p***	***z*-ratio**	***p***
Species-effect													
nye[†]–pun[‡]	broad[‖]	0.62	0.809	0.11	0.993	–1.43	0.918	0.47	0.886	–0.60	0.818	0.20	1.000
	red[¶]	–0.69	0.772	1.60	0.245	–0.24	1.000	0.21	0.977	0.80	0.705	–0.18	1.000
nye–sau[§]	broad	0.47	0.884	–1.30	0.393	–1.22	1.000	–0.79	0.712	–0.74	0.741	1.27	0.816
	red	–0.83	0.682	**–2.43**	**0.040**	0.45	1.000	**–2.41**	**0.043**	–2.21	0.069	**3.43**	**0.004**
pun–sau	broad	–0.12	0.991	–1.41	0.336	0.26	1.000	–1.20	0.455	–0.15	0.988	1.12	0.816
	red	–0.14	0.990	**–3.98**	**0.000**	0.62	1.000	**–2.47**	**0.036**	**–2.99**	**0.008**	**3.35**	**0.004**
Developmental treatment effect													
broad–red	pun	0.67	0.504	**1.97**	**0.049**	–0.85	0.396	0.99	0.321	1.18	0.236	–1.48	0.138
	nye	**2.15**	**0.031**	0.54	0.593	**–2.15**	**0.032**	1.28	0.200	–0.21	0.832	–1.14	0.254
	sau	0.62	0.533	–0.49	0.626	–0.60	0.549	–0.51	0.612	–1.58	0.115	0.83	0.409

[*]: group within which contrast is assessed
[†]: *Pundamilia* sp. '*nyererei*-like'
[‡]: *Pundamilia* sp. '*pundamilia*-like'
[§]: *Haplochromis sauvagei*
[‖]: broad spectrum developmental light treatment
[¶]: red-shifted developmental light treatment

Table S3. Results of post-hoc tests on the effect of species and developmental light treatment on visual gene expression. Significant differences ($p < 0.05$) are marked in bold.

		sws2ab / rh2a		***lws / rh2a***		***cyp27c1***	
**Contrast**	**Group[*]**	***t*-ratio**	***p***	***t*-ratio**	***p***	***t*-ratio**	***p***
Species-effect							
nye[†]–pun[‡]	broad[‖]	–2.27	0.065	**–2.88**	**0.014**	2.24	0.073
	red[¶]	**–5.37**	**0.000**	**–4.03**	**0.000**	**2.61**	**0.029**
nye–sau[§]	broad	0.25	0.965	0.94	0.619	1.93	0.140
	red	–0.56	0.844	0.16	0.986	1.59	0.256
pun–sau	broad	**2.47**	**0.041**	**3.69**	**0.001**	0.05	0.998
	red	**4.96**	**0.000**	**4.34**	**0.000**	–1.19	0.463
Developmental treatment effect							
broad–red	pun	–1.83	0.072	–0.90	0.369	1.34	0.184
	nye	**2.15**	**0.035**	0.54	0.589	0.11	0.910
	sau	1.28	0.205	–0.37	0.714	–0.16	0.871

[*]: group within which contrast is assessed
[†]: *Pundamilia* sp. '*nyererei*-like'
[‡]: *Pundamilia* sp. '*pundamilia*-like'
[§]: *Haplochromis sauvagei*
[‖]: broad spectrum developmental light treatment
[¶]: red-shifted developmental light treatment

Table S4. Results of post-hoc tests on the effect of species and developmental light treatment on the intercept, slope and tipping point of the dorsoventral axis response to blue and red light, with visual gene expression included in the models. Significant differences ($p < 0.05$) are marked in bold. Contrasts that were significant in the models without visual gene expression included are coloured red (Table S2).

		Blue test light						Red test light					
		Intercept		Slope		Tipping point		Intercept		Slope		Tipping point	
Contrast	Group[*]	*z*-ratio	*p*	*z*-ratio	*p*	*z*-ratio	*p*	*z*-ratio	*p*	*z*-ratio	*p*	*z*-ratio	*p*
Species-effect													
nye[†]–pun[‡]	broad[ǀ]	1.34	0.375	0.93	0.619	–1.18	1.000	1.00	0.575	0.87	0.660	–0.49	1.000
	red[¶]	0.68	0.777	0.48	0.879	–0.45	1.000	1.13	0.493	–0.23	0.970	0.07	1.000
nye–sau[§]	broad	0.19	0.979	–0.18	0.981	–0.40	1.000	–0.49	0.878	0.52	0.860	0.14	1.000
	red	0.48	0.879	–1.39	0.345	–0.22	1.000	–1.54	0.273	–2.18	0.074	**2.86**	**0.021**
pun–sau	broad	–1.01	0.568	–1.00	0.578	0.65	1.000	–1.29	0.403	–0.31	0.948	0.57	1.000
	red	–0.29	0.956	–1.70	0.206	0.29	1.000	**–2.56**	**0.028**	–1.58	0.256	**3.09**	**0.012**
Developmental treatment effect													
broad–red	pun	0.63	0.526	1.26	0.207	–0.55	0.581	1.15	0.249	0.14	0.886	–1.19	0.236
	nye	1.00	0.317	1.40	0.163	–1.07	0.286	0.56	0.573	1.40	0.161	–1.42	0.157
	sau	1.24	0.215	0.20	0.840	–0.93	0.351	–0.57	0.569	–1.27	0.204	1.35	0.177

[*]: group within which contrast is assessed
[†]: *Pundamilia* sp. '*nyererei*-like'
[‡]: *Pundamilia* sp. '*pundamilia*-like'
[§]: *Haplochromis sauvagei*
[ǀ]: broad spectrum developmental light treatment
[¶]: red-shifted developmental light treatment

Table S5. List of fish used in experiments.

Fish ID	Species	Developmental light treatment	Sex	Clutch[*]	Clutch mother[*]	Clutch father[*]	Generation[†]	Age at first trial (days)	Sampling delay (days)[‡]
2770	nye[§]	broad-spectrum	m	12n1	nnnnnn1	nnnnnn1	3.5	880	13
4092	nye	broad-spectrum	m	8n1	nnnn8	nnnn8	3	339	28
4108	nye	broad-spectrum	m	8n2	nnnn8	nnnn8	3	357	16
4118	nye	broad-spectrum	m	8n3	nnnn8	nnnn8	3	311	13
4124	nye	broad-spectrum	m	8n4	nnnn8	nnnn8	3	321	16
4459	nye	broad-spectrum	f	8n5	nnnn8	nnnn8	3	319	16
4238	nye	broad-spectrum	m	nnnn1	nn18	nn19	2	1617	5
714	nye	broad-spectrum	m	nnnn7	nn24	nn27	2	1278	108
2942	nye	broad-spectrum	f	nnnn8	nn15	nn28	2	577	105
2941	nye	broad-spectrum	m	nnnn8	nn15	nn28	2	556	182
3894	nye	broad-spectrum	m	nnnnnn1	nn24	nnnn6	2.5	414	91
663	nye	broad-spectrum	m	nnnnnn1	nn24	nnnn6	2.5	825	98
671	nye	broad-spectrum	m	nnnnnn1	nn24	nnnn6	2.5	1116	6
639	nye	broad-spectrum	m	nnnnnn2	nn24	nnnn6	2.5	861	192
3634	nye	broad-spectrum	m	nnnnnn3	nnnn4	nn27	2.5	470	180
4069	nye	broad-spectrum	m	nnnnnn5	nnnn4	nn27	2.5	611	15
4100	nye	red-shifted	m	8n1	nnnn8	nnnn8	3	340	29
4113	nye	red-shifted	m	8n2	nnnn8	nnnn8	3	359	16
4121	nye	red-shifted	m	8n3	nnnn8	nnnn8	3	312	29
4135	nye	red-shifted	m	8n4	nnnn8	nnnn8	3	322	17
4361	nye	red-shifted	f	8n5	nnnn8	nnnn8	3	320	17
4362	nye	red-shifted	f	8n5	nnnn8	nnnn8	3	334	9
2511	nye	red-shifted	f	nnnn4	nn15	nn3	2	1274	251
2515	nye	red-shifted	f	nnnn4	nn15	nn3	2	1303	223
2503	nye	red-shifted	m	nnnn6	nn	nn3	2	1269	231
2977	nye	red-shifted	m	nnnn8	nn15	nn28	2	578	209
2978	nye	red-shifted	m	nnnn8	nn15	nn28	2	818	7
1047	nye	red-shifted	m	nnnnnn2	nn24	nnnn6	2.5	841	40
3687	nye	red-shifted	m	nnnnnn3	nnnn4	nn27	2.5	373	78
3716	nye	red-shifted	m	nnnnnn4	nnnn4	nn27	2.5	400	167
3717	nye	red-shifted	m	nnnnnn4	nnnn4	nn27	2.5	651	13
3718	nye	red-shifted	m	nnnnnn4	nnnn4	nn27	2.5	676	12
3719	nye	red-shifted	m	nnnnnn4	nnnn4	nn27	2.5	683	26
4085	nye	red-shifted	m	nnnnnn5	nnnn4	nn27	2.5	598	8
472	pun[ǀ]	broad-spectrum	m	pppp11	pp11	pp13	2	1317	203
633	pun	broad-spectrum	m	pppp11	pp11	pp13	2	1557	15
817	pun	broad-spectrum	f	pppp12	pp11	pp9	2	832	98
706	pun	broad-spectrum	m	pppp13	pp11	pp13	2	1120	10
1433	pun	broad-spectrum	m	pppp14	pp11	pp9	2	1086	12
1962	pun	broad-spectrum	f	pppp15	pp17	pp15	2	781	106
1842	pun	broad-spectrum	f	pppp16	pp6	pp15	2	668	180
1984	pun	broad-spectrum	m	pppp17	pp11	pp16	2	993	12
2252	pun	broad-spectrum	f	pppp18	pp11	pp16	2	976	7
142	pun	broad-spectrum	m	pppp2	pp11	pp9	2	1680	190
834	pun	broad-spectrum	m	pppp8	pp11	pp9	2	1623	12
715	pun	broad-spectrum	f	pppppp1	pp16	pppp9	2.5	951	53
480	pun	broad-spectrum	m	pppppp2	pp16	pppp9	2.5	948	48

Fish ID	Species	Developmental light treatment	Sex	Clutch*	Clutch mother*	Clutch father*	Generation†	Age at first trial (days)	Sampling delay (days)‡
1194	pun	broad-spectrum	m	pppppp3	pp11	pppp1	2.5	979	19
1469	pun	broad-spectrum	f	pppppp4	pp11	pppp1	2.5	916	51
2929	pun	broad-spectrum	m	pppw2	pp11	pw	1.5	835	13
2501	pun	red-shifted	f	pppp10	pp11	pp13	2	1336	83
2500	pun	red-shifted	m	pppp11	pp11	pp13	2	1618	17
1660	pun	red-shifted	f	pppp12	pp11	pp9	2	1838	166
953	pun	red-shifted	f	pppp13	pp11	pp13	2	1107	16
1394	pun	red-shifted	m	pppp14	pp11	pp9	2	1084	29
2299	pun	red-shifted	m	pppp15	pp17	pp15	2	795	230
1884	pun	red-shifted	m	pppp16	pp6	pp15	2	920	13
2006	pun	red-shifted	f	pppp17	pp11	pp16	2	685	252
2277	pun	red-shifted	m	pppp18	pp11	pp16	2	976	27
1043	pun	red-shifted	m	pppp3	pp11	pp9	2	1768	82
2521	pun	red-shifted	m	pppp4	pp11	pp10	2	1573	104
4239	pun	red-shifted	m	pppp8	pp11	pp9	2	1582	9
974	pun	red-shifted	f	pppppp1	pp16	pppp9	2.5	1045	9
1005	pun	red-shifted	m	pppppp2	pp16	pppp9	2.5	937	82
1247	pun	red-shifted	f	pppppp3	pp11	pppp1	2.5	1057	9
1246	pun	red-shifted	m	pppppp3	pp11	pppp1	2.5	980	9
1452	pun	red-shifted	m	pppppp4	pp11	pppp1	2.5	1021	15
2937	pun	red-shifted	m	pppw2	pp11	pw	1.5	833	13
2203	sau¶	broad-spectrum	m	rrrr10	rrrw2	rr3rr4	2.75	981	10
1741	sau	broad-spectrum	m	rrrr11	rr1rr2	rr3rr4	3	901	8
1690	sau	broad-spectrum	f	rrrr13	rrrw1	rr3rr4	2.75	612	98
1678	sau	broad-spectrum	m	rrrr13	rrrw1	rr3rr4	2.75	786	53
2053	sau	broad-spectrum	f	rrrr14	rrrw2	rr3rr4	2.75	599	21
3183	sau	broad-spectrum	m	rrrr25	rr1rr2	rr3rr4	3	721	6
3365	sau	broad-spectrum	m	rrrr26	rrrw1	rr3rr4	2.75	767	12
3385	sau	broad-spectrum	m	rrrr27	rr1rr2	rr3rr4	3	465	112
3386	sau	broad-spectrum	m	rrrr27	rr1rr2	rr3rr4	3	692	6
3468	sau	broad-spectrum	m	rrrr29	rr1rr2	rr3rr4	3	459	113
3750	sau	broad-spectrum	m	rrrr35	rr1rr2	rr3rr4	3	654	10
3765	sau	broad-spectrum	m	rrrr36	rrrw2	rr3rr4	2.75	690	16
1626	sau	broad-spectrum	f	rrrr4	rr1rr2	rr3rr4	3	712	180
1627	sau	broad-spectrum	f	rrrr4	rr1rr2	rr3rr4	3	734	105
2146	sau	broad-spectrum	m	rrrr8	rr1rr2	rr3rr4	3	994	6
2235	sau	red-shifted	m	rrrr10	rrrw2	rr3rr4	2.75	954	29
1753	sau	red-shifted	f	rrrr11	rr1rr2	rr3rr4	3	627	252
1754	sau	red-shifted	f	rrrr11	rr1rr2	rr3rr4	3	656	210
2082	sau	red-shifted	m	rrrr14	rrrw2	rr3rr4	2.75	861	9
1816	sau	red-shifted	f	rrrr16	rr1rr2	rr3rr4	3	841	11
3030	sau	red-shifted	m	rrrr1b	rr1rr2	rr3rr4	3	904	82
2656	sau	red-shifted	m	rrrr20	rr1rr2	rr3rr4	3	827	8
3159	sau	red-shifted	m	rrrr25	rr1rr2	rr3rr4	3	810	13
3354	sau	red-shifted	m	rrrr26	rrrw1	rr3rr4	2.75	488	113
3451	sau	red-shifted	m	rrrr29	rr1rr2	rr3rr4	3	720	15
3481	sau	red-shifted	f	rrrr30	rr1rr2	rr3rr4	3	447	204
3555	sau	red-shifted	m	rrrr32	rrrw2	rr3rr4	2.75	422	103
3759	sau	red-shifted	m	rrrr35	rr1rr2	rr3rr4	3	654	9
3778	sau	red-shifted	f	rrrr36	rrrw2	rr3rr4	2.75	658	27
3779	sau	red-shifted	m	rrrr36	rrrw2	rr3rr4	2.75	658	27
2173	sau	red-shifted	m	rrrr8	rr1rr2	rr3rr4	3	911	9

*: Identifiers of clutch of the fish and the clutch of the parents refer to an (arbitrary) identifier of the clutch into which the fish and the parents of the fish were born in.
†: Number of generations since the wild-caught parents. Generation was calculated as the mean of the generations of both parents plus one, where the generation of wild-caught fish was set to zero.
‡: Number of days between the last trial and the sampling of the fish' eyes.
§: *Pundamilia* sp. '*nyererei*-like'
ǀ: *Pundamilia* sp. '*pundamilia*-like'
¶: *Haplochromis sauvagei*

Table S6. All gene-specific primers used in the current study to quantify gene expression in qPCR assays. References refer to the first study in which the primer was used.

Amplicon	Primer	Direction	Sequence (5'–3')
sws2b	sws2b_f1[1]	forward	GCGCTGCACTTCCACCTC
	sws2b_r2[2]	reverse	GGCCACAGGAACACTGCAT
sws2a	sws2a_f2[2]	forward	CAAGATYGAAGGTTTCATGGTA
	sws2a_r2[2]	reverse	CGCTCGAAAGCTATCACAGC
rh2a	rh2a_f2[1]	forward	TTCTGTGCWATTGAGGGATTC
	rh2a_r1[1]	reverse	CCAGGACAACAAGTGACCAGAG
lws	lws_f1[1]	forward	CTGTGCTACCTTGCTGTGTGG
	lws_r1[1]	reverse	GCCTTCTGGGTTGACTCTGACT
cyp27c1	cyp27c1_f1[3]	forward	GATGACTTGGTAGTTGGTGGA
	cyp27c1_r1[3]	reverse	GAACTTCTGAATGAGCCTTATGAG
ldh1	ldh1_f1[4]	forward	TTGGAGGTTTTAAGGAAAAGG
	ldh1_r1[4]	reverse	CAGGAACAAGGTGACGGTGGT
gadph2	gadph2_f1[3]	forward	TGTCTTCCAGTGTATGAAGCC
	gadph2_r1[3]	reverse	GGTCGTATTTGTCCTCATTAACTC
β-actin	bactin_f1[5]	forward	AATCGTGCGTGACATCAAGG
	bactin_r1[5]	reverse	AGTATTTACGCTCAGGTGGG

References: [1]: Carleton & Kocher (2001); [2]: Wright *et al.* (2019); [3]: Wilwert *et al.* (2022); [4]: Torres-Dowdall *et al.* (2017); [5]: Jin *et al.* (2013)

Table S7. Samples sizes of groups for which gene expression data complying with our qPCR quality control criteria could be obtained.

Species	Developmental light treatment	*sws2ab / rh2a*	*lws / rh2a*	*cyp27c1*
Pundamilia sp. '*pundamilia*-like'	broad-spectrum	14	14	10
	red-shifted	14	14	11
Pundamilia sp. '*nyererei*-like'	broad-spectrum	14	15	8
	red-shifted	17	17	7
Haplochromis sauvagei	broad-spectrum	13	12	7
	red-shifted	16	16	7

Table S8. Random effect structure of models describing the dorsoventral axis angle under blue and red test light.

		Variance	
Group	Variable*	Blue test light	Red test light
behavioural trial	intercept	11.0	8.64
behavioural trial	relative intensity†	0.471	0.640
individual	intercept	0.0315	0.148
individual	relative intensity	0.509	0.514
clutch	intercept	0.138	< 0.0001
species	first behavioural trial	0.156	< 0.0001
species	second behavioural trial	0.0770	0.0840
species	third behavioural trial	0.0420	0.0899
control light position	intercept	1.23	0.755
sex	intercept	0.0112	0.0563
residual variance	—	17.5	17.5

*: indicates whether the factor was included as a random intercept (*intercept*) or a random slope, and what dependent variable was used as the random slope

†: log_e-transformed relative intensity of test over control light

Table S9. Random effect structure of models describing the visual gene expression patterns.

	Variance		
Group	*sws2ab / rh2a*	*lws / rh2a*	*cyp27c1*
clutch	0.0678	0.000	0.000
sex	0.0318	0.000	0.0377
residual variance	0.328	0.569	0.325

Biology Open • Supplementary information

Table S10. Random effect structure of models describing the dorsoventral axis angle under blue and red test light, with visual gene expression included in the models.

Group	Variable[*]	Variance	
		Blue test light	Red test light
behavioural trial	intercept	9.92	7.40
behavioural trial	relative intensity[†]	0.578	0.516
individual	intercept	0.380	0.381
individual	relative intensity	0.266	0.284
clutch	intercept	< 0.0001	< 0.0001
species	first behavioural trial	< 0.0001	0.0995
species	second behavioural trial	0.897	0.246
species	third behavioural trial	0.0422	0.0332
control light position	intercept	0.915	0.504
sex	intercept	< 0.0001	< 0.0001
residual variance	—	17.6	18.1

[*]: indicates whether the factor was included as a random intercept (*intercept*) or a random slope, and what dependent variable was used as the random slope
[†]: log_e-transformed relative intensity of test over control light

References

Carleton, K. L. and Kocher, T. D. (2001). Cone opsin genes of African cichlid fishes: tuning spectral sensitivity by differential gene expression. *Mol. Biol. Evol.* **18**, 1540–1550.

Jin, Y. H., Park, J. W., Kim, J.-H. and Kwon, J. Y. (2013). The expression pattern of melatonin receptor 1a gene during early life stages in the Nile tilapia (*Oreochromis niloticus*). *Dev. Reprod.* **17**, 45–53.

Maan, M. E., Seehausen, O. and Groothuis, T. G. G. (2017). Differential survival between visual environments supports a role of divergent sensory drive in cichlid fish speciation. *Am. Nat.* **189**, 78–85.

Torres-Dowdall, J., Pierotti, M. E. R., Härer, A., Karagic, N., Woltering, J. M., Henning, F., Elmer, K. R. and Meyer, A. (2017). Rapid and parallel adaptive evolution of the visual system of neotropical Midas cichlid fishes. *Mol. Biol. Evol.* **34**, 2469–2485.

Wilwert, E., Etienne, R. S., Zande, L. and Maan, M. E. (2022). Contribution of opsins and chromophores to cone pigment variation across populations of Lake Victoria cichlids. *J. Fish Biol.* **101**, 365–377.

Wright, D. S., Meijer, R., Eijk, R., Vos, W., Seehausen, O. and Maan, M. E. (2019). Geographic variation in opsin expression does not align with opsin genotype in Lake Victoria cichlid populations. *Ecol. Evol.* **9**, 8676–8689.

Biology Open • Supplementary information
