# Supplementary material for: Correlating relative light sensitivity to retinal visual gene expression patterns in three African cichlid fish species
Source: Biol Open. 2026 Oct 12;15(10):bio062674. doi: 10.1242/bio.062674 (PMC13714874; doi:10.1242/bio.062674)
Supplement: Supplementary file 2 [file biolopen-15-062674_review_history.pdf]

Biology Open

# Correlating relative light sensitivity to retinal visual gene expression patterns in three African cichlid fish species

Gerrit Potkamp, Elena Zhemchuzhnikova, Felicity van Geresteijn, Joyce A. Luijben, Floor A. Munnik, Thomas A. Veldhuis, Martine E. Maan, Louis van de Zande and Rampal S. Etienne

10.1242/bio.062674

**Editor**: Valentina di Santo

**Review timeline**

| | |
|---|---|
| Original submission: | 1 May 2026 |
| Editorial decision: | 8 May 2026 |
| First revision received: | 31 August 2026 |
| Accepted: | 4 September 2026 |

---

**Original submission**

First decision letter

MS ID#: bio.062674

MS Title: Correlating relative light sensitivity to retinal visual gene expression patterns in three African cichlid fish species

Authors: Gerrit Potkamp; Elena Zhemchuzhnikova; Felicity van Geresteijn; Joyce A. Luijben; Floor A. Munnik; Thomas A. Veldhuis; Martine E. Maan; Louis van de Zande; Rampal S. Etienne

I have now reached a decision on the above manuscript.

The reviewer reports are shown at the bottom of this email.

**As you will see, the reviewers raised a number of substantial criticisms that prevent me from accepting the paper at this stage.**

They suggest, however, that a revised version might prove acceptable, if you can address their concerns. If you think that you can deal satisfactorily with the criticisms on revision, I would be pleased to see a revised manuscript. We would then return it to the reviewers.

At this stage, we also ask you to ensure your manuscript complies with our formatting guidelines. Provided you are able to fully address the referees' comments, we are positive about publication of your paper (we accept over 95% of revision submissions) and therefore hope you won't mind any extra work involved in reformatting your manuscript at this point.

Please upload both a 'clean' version of your Word file, along with a highlighted version clearly showing where you have made changes in the revised manuscript. Please avoid using 'Track changes' in Word files as these are lost in PDF conversion.

© 2026. Published by The Company of Biologists under the terms of the Creative Commons Attribution License (https://creativecommons.org/licenses/by/4.0/).

I should be grateful if you would also provide a point-by-point response detailing how you have dealt with the points raised by the reviewers in the 'Response to Reviewers' box. Please attend to all of the reviewers' comments. If you do not agree with any of their criticisms or suggestions please explain clearly why this is so.

Reviewer 1

Comments for the author

The central question of this manuscript is whether there is a link between opsin gene expression and visual sensitivity. This relationship is often used to try to understand evolution and selection of the visual system in either different aquatic environments or during the transition from an aquatic to a terrestrial mode of living, particularly in fish species. However, directly quantifying the link between opsin gene expression and visual sensitivity has proven difficult.

Potkamp et al. propose in their study to use the dorsal light response as a behavioural proxy to quantify relative visual sensitivity to blue and red light in three cichlid species. They, in addition, used individuals that were raised under different light conditions. Overall, the authors report subtle but significant differences in sensitivity among species and treatments but fail to observe an alignement of sensitivity with group-level patterns of opsin gene expression. However, at the individual level, correlations between opsin gene expression ratios and visual sensitivity are observed. The main claim of the study is that opsin gene expression alone is insufficient to explain variation in visual sensitivity and that downstream neural processes should be more taken into account.

The strengths of the study include the combination of a behavioural assay with gene expression analyses across three species and several light treatments. The use of the dorsal light response is valuable as it could avoid some pitfalls associated with more complex behaviours. The statistical modeling is comprehensive with appropriate use of mixed-effects models and does a good job at addressing potential issues associated for example with age or sex biases.

Overall, the study is important for the field of evolution of visual system and the conclusion are well supported with the existing data set. Yet the point 1 below, relating to the delay in sampling, is a major point to address. My understanding of the study is that with the current dataset, the conclusion holds, but that the dataset is not ideal and the conclusion could be different with a more controlled dataset.

Points that require attention:

1. While the statistical models try to account for differences into the gene expression measurement time after behavioural trials, the timing of measurements happened between days and months after the behavioural test. Fig. S4 already shows a decay in relation with sampling delay. This delay could in fact obscure group-level effects and therefore the main conclusion of the study as well as the title may be invalid. Time-matched gene expression analysis and shortly after the behavioural tests would be needed to conclude firmly. Therefore, this main gap should be more clearly stated in the abstract and the title.

2. The experimental setup with LED and filters appears appropriate and light intensity seems to be in range with natural habitat light intensities, although this is not clearly explained. However, LED flickering or non-uniformity of the filters, particularly at different light intensities could affect the claim that the assay is a direct quantification of visual sensitivity. It is unclear how these issues were taken into account or if they were measured?

3. The sample size for cyp27c1 is smaller than for the other genes studied and this is not mentionned in the text. I suppose some of the data were excluded because of the lower expression of cyp27c1, but it would be important to check that the excluded data were random or biased.

Reviewer 2

© 2026. Published by The Company of Biologists under the terms of the Creative Commons Attribution License (https://creativecommons.org/licenses/by/4.0/). 

Comments for the author

This is a well-conducted and generally well-presented study that tests the correlation between retinal photopigment gene-expression and behavioral light sensitivity expressed in the dorsal light response (used as a behavioral assay)

My expertise is in general visual function, psychometrics, and behavior, and not in fish biology or retinal genetics/molecular biology.

General

I trust the authors' statement that the literature in this field has been using the (relatively easily-measured) retinal gene expression as a proxy for visual function and thereby as an explanatory factor for the evolution of visual function in this class of fish. As a neuroscientist who primarily studies central visual processing, it is amusing to see that just as "brain scientists" often tend to disregard the substantial retinal processing, it appears that retinal scientists similarly put lump together processing beyond the photoreceptor into a generic "processing of signals in the brain" block. I suggest that the Reviewers expand on this, since this is also their main point, and keep the Data Processing Inequality from information theory in mind --- function that is lost at the photoreceptor level cannot be regained, but function that is retained at the photoreceptor level can definitely be lost at higher levels. In particular, since the variation sought to be explained is across-species and across-condition rather than within-individual, it is quite natural that there may be (adaptive) post-photoreceptor differences that affect behavior and thereby genetic fitness/evolutionary viability. I would also recommend that the authors emphasize a bit more (carefully) that photopigment variations do still affect within-individual function. For example, "This discrepancy shows that the assumption that visual gene expression patterns are the main determinants of visual sensitivity needs reconsideration" could be subtly rewritten to use the phrase "across-group variations in visual sensitivity", and again keeping the data processing inequality in mind.

Major concerns
--------------

My major concerns are with the text of the Results section and the statistical approach. I am unable to evaluate if/how much any of these concerns (will) affect the conclusions of the study; however, in my reading at least, there are several aspects that can be substantially improved, or at least addressed directly to assuage these concerns.

1. The model-selection procedure appears to be a data-driven stepwise process involving both random- and fixed-effect selection: this approach is problematic (e.g. see books and papers notably by Frank Harrell and many others) and inflates significance. Using a theoretically-guided model with penalized regression strategies in case of non-identifiability issues is often recommended. The authors should explain their modeling strategy in more detail -- though the Reviewer has no experience with the conventions in this field or with the data-quality from fish in this task, the model appears over-specified, considers a lot of factors (given the number of data points) and then discards several by testing on the same dataset that the significance of the Results are reported from, which leads to issues with "double-dipping", "data-dredging" etc.

2. The authors should justify random effects based primarily on the sampling design, clarify whether fixed-effect comparisons were pre-specified, and consider reporting results from a full biologically motivated model or a small predefined candidate set rather than relying on iterative selection. The random-effects structure appears over-specified and is difficult to justify from the description. Several variables listed as random effects, such as sex and light position, seem more naturally treated as fixed effects, since they have only a small number of interpretable levels and the authors may wish to estimate their systematic influence. In particular, it is unclear why sex is modeled as a random intercept rather than a fixed effect. With only two biological categories, sex does not seem to represent a random sample from a larger population of levels. The same concern applies to left/right light position. These predictors may be better modeled as fixed effects, or at least the rationale for treating them as random should be explained.

© 2026. Published by The Company of Biologists under the terms of the Creative Commons Attribution License (https://creativecommons.org/licenses/by/4.0/).

3. Figures are insufficiently described. E.g. there is no reference to Figure 3b in manuscript and I do not know what I am supposed to read from that, either from the figure caption or from the text.

4. The treatment of the "psychometric function" in Figure 3 (and the analysis in general) may be the norm for this sub-field, but conventionally (see e.g. textbook by Prins and Kingdom), sensitivity is reserved for the slope and the vertical position of the line (deviating from zero) reflects bias (y-axis is not 0 when relative intensity is 1). Sensitivity here is defined as how the turning-angle changes for a given change in light intensity. The metric the authors are using mixes these two, and probably should not be called "sensitivity" -- slopes and intercepts can be separately analyzed, and often are (at least in the vast literature on human psychophysics). The authors appear to indeed calculate and use slope and intercept, but only for 1 particular point (and where they pivot from using the x-value at y=0 to slopes and intercepts for no apparent reason) -- see 6. below.

5. In Figure 4, the Y-axis range is too large, and makes it almost impossible to see the actual content. In particular, it is very hard to connect the data in Figure 4 to the Results in Figure 5, which are presumably based on the same data as in Figure 4. For example, the biggest difference between the x-axis values for y=0 between the two treatments (black and orange) appears to be for "nyererei-like" in Figure 4, but that has one of the smallest differences in Figure 5. Some of the differences in Figure 5 appear to be too large given the data in Figure 4. It is not clear why the scale has been transformed for Figure 5, rather than use the same scale as in Figure 4. It also appears that the significance tests shown in Figure 5 are based on some comparison across all light intensities (and it is not clear how this is done) -- this also relates to point 5. right below.

a. Given the above, it is not clear from this brief description, how any of these statements are supported: "Blue light sensitivity, relative to the green control light, differed significantly between individuals from different developmental light treatments (Figs. 4-5; see supplemental information for details on model selection). In fish from the broad-spectrum developmental light treatment, the dorsoventral axis angle under blue test light was significantly larger (i.e., fish tilted more towards the blue test light or less towards the green control light) compared to fish from the red-shifted developmental light treatment across all relative light intensities (z-ratio = 2.75; p = 0.006). This indicates that development in the broad-spectrum light treatment increased the perceived relative intensity of blue light, compared to the red-shifted developmental light treatment (Fig. 5)".

i) How was the z-ratio/z-score calculated here and elsewhere across all light intensities ? See note about sensitivty in point 5. below.

b. It would also help a lot to have tickmarks on the axes for all the plots in Figure 4 to make them easier to read.

6. It is not clear why the authors switch to using slopes and intercepts in the excerpt below. In the final sentence here for red stimulus light here, it is not clear which metric is being used. How were these calculations of slope and intercept averaged across the different values of the other factors ? "Relative sensitivity to blue light also differed significantly between species (Figs. 4-5). While the intercept of the response of the dorsoventral axis to relative light intensity did not vary significantly between species, as it did for fish from different light treatments (i.e., the dorsoventral axis of all species did not differ equally across all relative light intensities), the slope of the response was significantly higher in Haplochromis sauvagei compared to Pundamilia sp. 'pundamilia-like' (z-ratio = 3.60; p = 0.001), indicating that relative blue light sensitivity is higher in H. sauvagei (i.e., the dorsoventral axis angle equals zero for a lower relative light intensity in H. sauvagei compared to Pundamilia sp. 'pundamilia-like'). A significant species difference was also found in relative red light sensitivity (Fig. 4). With red stimulus light, Haplochromis sauvagei had a significantly higher dorsoventral axis angle than Pundamilia sp. nyererei-like' (z-ratio = 2.67; p = 0.021)."

7. The description of Figure 6 in the Results proceeds under the assumption that the metric of x@y=0 is the suitable one to measure sensitivity. Does the analysis change if the slope is used to compare the patterns in Figure 6 with the measure of light sensitivity ?

© 2026. Published by The Company of Biologists under the terms of the Creative Commons Attribution License (https://creativecommons.org/licenses/by/4.0/).

8. I doubt that this argument is correct as stated. This could be defensible if "explained" is replaced by "fully explained". Even so, with correlated predictors, fit quality may significantly improve even if not individual factor's effect is "statistically significant", depending on how the test is done. "If group-level variation in relative light expression could have been explained by group-level differences in visual gene expression patterns, species- and developmental light treatment-effects would not be statistically significant in a model that relates gene expression to relative light sensitivity."

9. Figure 7 is important for many reasons, since it shows an expected effect of opsin gene expression levels on visual function within individuals. The caption to Figure 7 could indicate that these plots are estimated within-individuals. This result is, in this Reviewer's opinion, . It appears from the Discussion that this effect has been tested and reported on before in other species, and this paper adds to that data. It is not surprising that the treatment and species level differences that the authors are interested in do not show clear correlations, given the multiple possibilities for adaptive changes in the rest of the pathway from gene expression to behavior -- the authors do clearly mention this, but the Discussion could be improved to bring this point to the forefront.

10. "The observed trend between opsin expression ratio and dorsoventral axis angle was strongest for individuals with a short delay between behavioural trials and sampling of the retinal tissue (Fig. S4)." - The authors are commendably transparent about the potential impact of the delay between the behavioral assay and the sampling of the retinal tissue. However, this result suggests that there was indeed a meaningful impact of this delay on their data. Would the group-level results change if only results from short-delays were used ?

Minor concerns
---------------

The manuscript, outside of the Results section, and especially the Methodology is generally very well-written.

Reviewer's Responses to Questions

**Experimental quality**
Are experiments performed using appropriate methods that will answer the question (or test the hypothesis or support the observations) posed by the authors? Is the right tool used for the job?
*If 'No', please indicate reasons in Comments for Author box below.*
Reviewer #1:

- Yes

Reviewer #2:

- Yes

---

Were the data analyzed using appropriate statistical tests?
*If 'No', please indicate reasons in Comments for Author box below.*
Reviewer #1:

- Yes

Reviewer #2:

- No

---

**Reproducibility**
Were experiments performed using adequate number of biological replicates?
*If 'No', please indicate reasons in Comments for Author box below.*
Reviewer #1:

- Yes

© 2026. Published by The Company of Biologists under the terms of the Creative Commons Attribution License (https://creativecommons.org/licenses/by/4.0/).

Reviewer #2:

- Yes

---

Is there sufficient raw data to assess the rigor of the analysis?
*If 'No', please indicate reasons in Comments for Author box below.*
Reviewer #1:

- Yes

Reviewer #2:

- Yes

---

Does the methods section provide sufficient detail to permit reproducibility?
*If 'No', please indicate reasons in Comments for Author box below.*
Reviewer #1:

- Yes

Reviewer #2:

- Yes

---

**Completeness**
Are the manuscript's conclusions supported by the data?
*If 'No', please indicate reasons in Comments for Author box below.*
Reviewer #1:

- Yes

Reviewer #2:

- No

---

**Scholarship**
Do the authors cite and discuss the merits of data that would argue for and against their conclusion?
*If 'No', please indicate reasons in Comments for Author box below.*
Reviewer #1:

- Yes

Reviewer #2:

- Yes

---

Does the manuscript title & abstract accurately reflect the contents of the manuscript, without hyperbole?
*If 'No', please indicate reasons in Comments for Author box below.*
Reviewer #1:

- No

Reviewer #2:

- Yes

---

**First revision**

Author response to reviewers' comments

Dear prof. dr. Di Santo,

© 2026. Published by The Company of Biologists under the terms of the Creative Commons Attribution License (https://creativecommons.org/licenses/by/4.0/).

Thank you for allowing us to revise our manuscript for publication in Biology Open. Below you find a detailed list of how we revised our manuscript in the light of the reviewers' comments. With these revisions, we hope to have alleviated the reviewer's concerns and hope you can consider our manuscript for publication.

On behalf of all authors,

Gerrit Potkamp

**Reviewer 1:**

***Comment 1**. While the statistical models try to account for differences into the gene expression measurement time after behavioural trials, the timing of measurements happened between days and months after the behavioural test. Fig. S4 already shows a decay in relation with sampling delay. This delay could in fact obscure group-level effects and therefore the main conclusion of the study as well as the title may be invalid. Time-matched gene expression analysis and shortly after the behavioural tests would be needed to conclude firmly. Therefore, this main gap should be more clearly stated in the abstract and the title.*

**Reply:** reviewer 1 is correct in stating that the sampling delay could have obscured our conclusion on the lack of a group-level correlation between visual gene expression and visual sensitivity. However, contrary to our results on individual-level correlations gene expression and visual sensitivity (which decay with sampling delay), we do not have any indications in our data that a sampling delay may have obscured a potential group-level correlation. First, we have a wide range in sampling delay in our data, and gene expression patterns generally do not seem to change with an increasing sampling delay (which we now show in Fig. S10), suggesting that, at the group-level, visual gene expression is not different shortly after the behavioural trials compared to longer after the trials. We have added this analysis to the manuscript (see lines 386–387 in the clean, revised manuscript and Fig. S10).

Second, quite a lot is known already on visual gene expression patterns in the species included in our study (including measurements from previous studies in our lab (Wright *et al.*, 2020; Wilwert *et al.*, 2023), on the same species in the same developmental light treatments). The general pattern there is that *Pundamilia* sp. '*nyererei*-like' and *Haplochromis sauvagei* have similar gene expression patterns, while *Pundamilia* sp. '*pundamilia*-like' has different gene expression patterns (e.g., higher relative *lws* expression, lower *cyp27c1* expression). Our gene expression results are consistent with this pattern (Fig. 6). In our DLR data however, both *Pundamilia* species have similar relative sensitivities, different from *Haplochromis sauvagei* (especially to red light; Fig. 5). Therefore, given what is known about basal visual gene expression patterns in these cichlids, the sampling delay probably did not obscure a group-level correlation. We have added this argument to the manuscript (lines 397–400).

Still, due to the sometimes long sampling delays, the reviewer is correct that more time-matched gene expression data is needed to decisively conclude that there is no group-level correlation. We have therefore toned down our statements by making the following changes: a), we reformulated the title; b), we have mentioned the sampling delay more explicitly in the abstract; and c), throughout the text, we have more explicitly stated that time-matched gene expression data is needed to firmly conclude that there is no group-level correlation.

***Comment 2**. The experimental setup with LED and filters appears appropriate and light intensity seems to be in range with natural habitat light intensities, although this is not clearly explained. However, LED flickering or non-uniformity of the filters, particularly at different light intensities could affect the claim that the assay is a direct quantification of visual sensitivity. It is unclear how these issues were taken into account or if they were measured?*

**Reply:** we have now addressed the point of flickering in the methods (lines 172–173): because we used variation in voltage to change intensity of the LEDs (instead of pulse-width-modulation), lower intensities of test and control light would not cause flickering of the LEDs. Regarding potential non-uniformity of the filters, we measured light intensities at two locations in the tube in which the fish

© 2026. Published by The Company of Biologists under the terms of the Creative Commons Attribution License (https://creativecommons.org/licenses/by/4.0/).

was housed during the experimental trials. While the absolute intensity changed slightly depending on the position, the relative intensity of test over control light was relatively stable. We have updated Fig. S3 to include this data.

Finally, we compared the light intensity of light stimuli to the natural habitat light intensities, which are indeed in the same order of magnitude. We have added a line on this comparison (lines 180–182).

***Comment 3**. The sample size for cyp27c1 is smaller than for the other genes studied and this is not mentionned in the text. I suppose some of the data were excluded because of the lower expression of cyp27c1, but it would be important to check that the excluded data were random or biased.*

**Reply:** the reason why the sample size of *cyp27c1* is lower than for the opsin expression ratios is that for measuring the relative *cyp27c1* expression, data was needed for 4 genes in total (3 reference genes and *cyp27c1*), compared to only 2 or 3 genes for the opsin expression ratio (only the opsins included in the ratio, data from the reference genes was not needed to calculate this ratio). Because of our quality control criteria (lines 238–241), this resulted in a slightly lower sample size for *cyp27c1*. This did not introduce biases in the sample size, the missing data seemed to be randomly distributed across groups (we added lines 250–252 and Table S4 with this information).

***Reviewer 2:***

***Comment 1**: I trust the authors' statement that the literature in this field has been using the (relatively easily-measured) retinal gene expression as a proxy for visual function and thereby as an explanatory factor for the evolution of visual function in this class of fish. As a neuroscientist who primarily studies central visual processing, it is amusing to see that just as "brain scientists" often tend to disregard the substantial retinal processing, it appears that retinal scientists similarly put lump together processing beyond the photoreceptor into a generic "processing of signals in the brain" block. I suggest that the Reviewers expand on this, since this is also their main point, and keep the Data Processing Inequality from information theory in mind --- function that is lost at the photoreceptor level cannot be regained, but function that is retained at the photoreceptor level can definitely be lost at higher levels. In particular, since the variation sought to be explained is across-species and across-condition rather than within-individual, it is quite natural that there may be (adaptive) post-photoreceptor differences that affect behavior and thereby genetic fitness/evolutionary viability. I would also recommend that the authors emphasize a bit more (carefully) that photopigment variations do still affect within-individual function. For example, "This discrepancy shows that the assumption that visual gene expression patterns are the main determinants of visual sensitivity needs reconsideration" could be subtly rewritten to use the phrase "across-group variations in visual sensitivity", and again keeping the data processing inequality in mind.*

**Reply:** we agree with the reviewer that we were too general in our remarks regarding post-photoreceptor processes. The mechanisms related to these processes are somewhat outside our area of expertise.

Additionally, very little is known about post-photoreceptor processes in cichlids, unfortunately. However, the reviewer is correct in that this relates to our main conclusions. We have therefore added a paragraph to the discussion expanding on this point (consulting an evolutionary neuroscientist from our institute), including an example of such processes in mice (lines 476–495). We thank the reviewer in pointing us towards the data processing inequality theory, which we used in our discussion.

We followed the reviewer's suggestion regarding emphasising the role of photopigment variation in within-individual function.

*Major concerns*

*--------------*

*My major concerns are with the text of the Results section and the statistical approach. I am*

© 2026. Published by The Company of Biologists under the terms of the Creative Commons Attribution License (https://creativecommons.org/licenses/by/4.0/).

*unable to evaluate if/how much any of these concerns (will) affect the conclusions of the study; however, in my reading at least, there are several aspects that can be substantially improved, or at least addressed directly to assuage these concerns.*

***Comment 1****. The model-selection procedure appears to be a data-driven stepwise process involving both random- and fixed-effect selection: this approach is problematic (e.g. see books and papers notably by Frank Harrell and many others) and inflates significance. Using a theoretically-guided model with penalized regression strategies in case of non-identifiability issues is often recommended. The authors should explain their modeling strategy in more detail -- though the Reviewer has no experience with the conventions in this field or with the data-quality from fish in this task, the model appears over-specified, considers a lot of factors (given the number of data points) and then discards several by testing on the same dataset that the significance of the Results are reported from, which leads to issues with "double-dipping", "data-dredging" etc.*

***Comment 2****. The authors should justify random effects based primarily on the sampling design, clarify whether fixed-effect comparisons were pre-specified, and consider reporting results from a full biologically motivated model or a small predefined candidate set rather than relying on iterative selection. The random- effects structure appears over-specified and is difficult to justify from the description. Several variables listed as random effects, such as sex and light position, seem more naturally treated as fixed effects, since they have only a small number of interpretable levels and the authors may wish to estimate their systematic influence. In particular, it is unclear why sex is modeled as a random intercept rather than a fixed effect. With only two biological categories, sex does not seem to represent a random sample from a larger population of levels. The same concern applies to left/right light position. These predictors may be better modeled as fixed effects, or at least the rationale for treating them as random should be explained.*

**Reply:** we recognise the concerns of reviewer 2 regarding our statistical modelling and agree with the risks related to data-dredging. The fish used in this study came from a heterogenous group of fish, and availability of fish was often limited (we have made this more explicit in the methods). As a result of this, there were several factors for which we needed to control using random effects, but not all these random effects contributed to the model.

To overcome the risks brought up by reviewer 2, we re-evaluated our statistical design and changed our approach. Following the suggestion, we selected the most important factors for which we needed to control using random effects, and included those in our model. To make this possible, we had to remove data from the fourth and fifth experimental trials that we did for some fish (14 trials in total), so that all fish in our dataset were subjected to exactly three experimental trials. Additionally, we simplified our fixed-effect structure (for example, by not including all possible interactions, which would not make sense with our limited sample size anyway: we removed for example an interaction between developmental light treatment and gene expression – we agree with the reviewer that our full model was over-specified given our sample size). We did also add some additional fixed effects, however, such as the absolute intensity of the control light. After that, we had a more limited subset of the most important fixed and random effects. In this revised approach, we considered this model our final model, and did not do any model comparisons. All of our statistical and post-hoc testing was done based on this pre-defined model.

Changing our approach in this way resulted in some statistical differences (some effects that were statistically significant in the old approach were not significant anymore and vice versa), but our main conclusion did not change with this new approach: interspecific differences in relative light sensitivity do not align with gene expression patterns, indicating that these conclusions are robust.

The reviewer mentions that some factors, such as sex or control light position, were treated in our models as random variables rather than fixed variables. We decided not to follow the reviewer's suggestion here, and kept these factors as random variables in our models. The reason why we treat sex as a random effect (instead of estimating the effect of sex by using it as a fixed effect) is that we did not have enough females in our dataset to reliably assess a sex-effect (Table S1).

Regarding the effect of control light position, our aim here was to only control for this

© 2026. Published by The Company of Biologists under the terms of the Creative Commons Attribution License (https://creativecommons.org/licenses/by/4.0/).

effect, and not assess the systematic effect of control light position. We deem a random effect more suitable in that case (despite there only being two levels for control light position).

With the revision of our statistical design, and the fact that our main conclusions did not change with the new design, we hope to have alleviated the reviewer's concerns regarding the statistics.

***Comment 3***. *Figures are insufficiently described. E.g. there is no reference to Figure 3b in manuscript and I do not know what I am supposed to read from that, either from the figure caption or from the text.*

**Reply:** we have made an explicit reference to figure 3A and 3B, and explained the message of these figures in the main text.

***Comment 4***. *The treatment of the "psychometric function" in Figure 3 (and the analysis in general) may be the norm for this sub-field, but conventionally (see e.g. textbook by Prins and Kingdom), sensitivity is reserved for the slope and the vertical position of the line (deviating from zero) reflects bias (y-axis is not 0 when relative intensity is 1). Sensitivity here is defined as how the turning-angle changes for a given change in light intensity. The metric the authors are using mixes these two, and probably should not be called "sensitivity" -- slopes and intercepts can be separately analyzed, and often are (at least in the vast literature on human psychophysics). The authors appear to indeed calculate and use slope and intercept, but only for 1 particular point (and where they pivot from using the x-value at y=0 to slopes and intercepts for no apparent reason) -- see 6. below.*

**Reply:** the reviewer is correct that in a psychometric function, slope of the response can be defined as the sensitivity to a stimulus. However, when using the dorsal light response to predict relative light sensitivity, the slope of the response is not the most informative measure. The dorsoventral axis under specific light stimuli does not only depend on the relative intensity of the light stimuli, but also on other factors. In the brain of the fish, information on light intensity is integrated with information on gravity (Pfeiffer, 1964). Additionally, other factors (such as the physiological state of the fish) play a role in the strength of the dorsal light response (i.e., how strong a fish tilts towards the test or control light; see Von Holst, 1948; Pfeiffer and Riegelbauer, 1978; Brodsky, 1999). We have added this argument to the manuscript (lines 102–107). The effect of such external factors is minimised by using the specific point where the dorsoventral axis angle equals 0°. We show this in Fig. S6, which we added to the manuscript in this revision. Additionally, we made the reasoning behind using the x-axis intercept (which has also been used in the few studies using a similar method before, see Silver, 1974; Powers, 1978; Muntz *et al.*, 1996) more clear in earlier the methods (lines 91–101).

However, we agree with the reviewer that the term 'sensitivity' may be confusing here, as this term could be used to describe the slope of the response as well (even though we don't expect correlations with visual gene expression for this 'sensitivity'). We have therefore introduced the term 'tipping point' to refer to the x-axis intercept. We have changed the term 'relative sensitivity' on the y-axes in Figs. 5 and 7 and in the supplemental figures to 'tipping point'. Additionally, we have introduced explicit statistical tests to test for differences in this tipping point. Wherever possible, we report on statistical differences of the intercept, slope and tipping point separately in the revision of this manuscript. We have added figures in the supplemental information on group-level differences in slope of the response (Fig. S8, see also point 6 of this reviewer below), and report on correlations between the slope of the response and visual gene expression (there are no such correlation, lines 435–437 & 446–447, Figs. S12; S14). Finally, in the methods, we have made it more explicit that we use the tipping point as a metric for visual sensitivity to the test light relative to the control light (lines 96–99: *"The relative intensity at which the dorsoventral axis angle equals 0° (i.e., the fish does not tilt towards the test or the control light), i.e., where a fish is assumed to perceive both stimuli as equally intense), serves as a measure for relative, visual sensitivity of the test light relative to the control light"*; line 258–259: *"We assessed differences in relative light sensitivity by comparing this tipping point among groups"*). With these changes, we hope to have removed the ambiguity related to the term 'sensitivity'.

***Comment 5***. *In Figure 4, the Y-axis range is too large, and makes it almost impossible to see*

© 2026. Published by The Company of Biologists under the terms of the Creative Commons Attribution License (https://creativecommons.org/licenses/by/4.0/).

*the actual content. In particular, it is very hard to connect the data in Figure 4 to the Results in Figure 5, which are presumably based on the same data as in Figure 4. For example, the biggest difference between the x-axis values for y=0 between the two treatments (black and orange) appears to be for "nyererei-like" in Figure 4, but that has one of the smallest differences in Figure 5. Some of the differences in Figure 5 appear to be too large given the data in Figure 4. It is not clear why the scale has been transformed for Figure 5, rather than use the same scale as in Figure 4. It also appears that the significance tests shown in Figure 5 are based on some comparison across all light intensities (and it is not clear how this is done) -- this also relates to point 5. right below.*

*a. Given the above, it is not clear from this brief description, how any of these statements are supported: "Blue light sensitivity, relative to the green control light, differed significantly between individuals from different developmental light treatments (Figs. 4-5; see supplemental information for details on model selection). In fish from the broad-spectrum developmental light treatment, the dorsoventral axis angle under blue test light was significantly larger (i.e., fish tilted more towards the blue test light or less towards the green control light) compared to fish from the red-shifted developmental light treatment across all relative light intensities (z-ratio = 2.75; p = 0.006). This indicates that development in the broad-spectrum light treatment increased the perceived relative intensity of blue light, compared to the red-shifted developmental light treatment (Fig. 5)".*

*i) How was the z-ratio/z-score calculated here and elsewhere across all light intensities ? See note about sensitivty in point 5. below.*
*b. It would also help a lot to have tickmarks on the axes for all the plots in Figure 4 to make them easier to read.*

**Reply:** we changed the limits of the y-axis in Fig. 4 to make the link to Fig. 5 clearer. We added the original Fig. 4 to the supplemental information. However, a large part of the confusion of the reviewer here is probably related to a mistake in our Fig. 5: the labels for *Pundamilia* sp. ‘*pundamilia*-like’ and *Pundamilia* sp. ‘*nyererei*-like’ in that figure were switched. The reason for that switch is that we used by mistake an incorrect, unordered factor level in R to create that figure, resulting in a different order of species in Fig. 5, compared to the other figures. The facet labels of the figures were edited in Adobe Illustrator to add the correct italics, where the difference in the order of the species was missed. We corrected our mistake in the revised version, which should address the confusion of the reviewer, which we thank for detecting this mistake!

Regarding tickmarks: we added tickmarks to all the plots in our figures.
*z*-scores were calculated using the R-packages emmeans and car.

***Comment 6****. It is not clear why the authors switch to using slopes and intercepts in the excerpt below. In the final sentence here for red stimulus light here, it is not clear which metric is being used. How were these calculations of slope and intercept averaged across the different values of the other factors ? "Relative sensitivity to blue light also differed significantly between species (Figs. 4-5). While the intercept of the response of the dorsoventral axis to relative light intensity did not vary significantly between species, as it did for fish from different light treatments (i.e., the dorsoventral axis of all species did not differ equally across all relative light intensities), the slope of the response was significantly higher in Haplochromis sauvagei compared to Pundamilia sp. 'pundamilia-like' (z-ratio = 3.60; p = 0.001), indicating that relative blue light sensitivity is higher in H. sauvagei (i.e., the dorsoventral axis angle equals zero for a lower relative light intensity in H. sauvagei compared to Pundamilia sp. 'pundamilia-like'). A significant species difference was also found in relative red light sensitivity (Fig. 4). With red stimulus light, Haplochromis sauvagei had a significantly higher dorsoventral axis angle than Pundamilia sp. nyererei-like' (z-ratio = 2.67; p = 0.021)."*

**Reply:** see our response to point 4, the changes implemented also address this point.

***Comment 7****. The description of Figure 6 in the Results proceeds under the assumption that the metric of x@y=0 is the suitable one to measure sensitivity. Does the analysis change if the slope is used to compare the patterns in Figure 6 with the measure of light sensitivity ?*

© 2026. Published by The Company of Biologists under the terms of the Creative Commons Attribution License (https://creativecommons.org/licenses/by/4.0/). 

**Reply:** we added this analysis to the manuscript, comparing the slopes of the response to light to gene expression patterns (see Figs. S8; S12; S14, and lines 403–404, 435–437 & 446–447): the conclusion does not change, there was no correlation with group-level variation in gene expression. See also our response to point 4 regarding the slope of the response vs. the x-intercept.

***Comment 8****. I doubt that this argument is correct as stated. This could be defensible if "explained" is replaced by "fully explained". Even so, with correlated predictors, fit quality may significantly improve even if not individual factor's effect is "statistically significant", depending on how the test is done. "If group-level variation in relative light expression could have been explained by group-level differences in visual gene expression patterns, species- and developmental light treatment-effects would not be statistically significant in a model that relates gene expression to relative light sensitivity."*

**Reply:** we agree with reviewer 2 that 'explained' should at least be replaced by 'fully explained'. We have however rewritten this paragraph to make our point here clearer (lines 410–418).

***Comment 9****. Figure 7 is important for many reasons, since it shows an expected effect of opsin gene expression levels on visual function within individuals. The caption to Figure 7 could indicate that these plots are estimated within-individuals. This result is, in this Reviewer's opinion, . It appears from the Discussion that this effect has been tested and reported on before in other species, and this paper adds to that data. It is not surprising that the treatment and species level differences that the authors are interested in do not show clear correlations, given the multiple possibilities for adaptive changes in the rest of the pathway from gene expression to behavior -- the authors do clearly mention this, but the Discussion could be improved to bring this point to the forefront.*

**Reply:** part of the reviewer's point was missing here, so we were not able to fully understand what the reviewer was referring to. We do however agree with the point that changes in the rest of the pathway towards behaviour are important and to be expected. We added a paragraph to the discussion related to this point, also including the reviewer's earlier suggestions regarding incorporating a section on post- photoreceptor processing and data processing inequality to the discussion (lines 476–495)

***Comment 10****. "The observed trend between opsin expression ratio and dorsoventral axis angle was strongest for individuals with a short delay between behavioural trials and sampling of the retinal tissue (Fig. S4)." - The authors are commendably transparent about the potential impact of the delay between the behavioral assay and the sampling of the retinal tissue. However, this result suggests that there was indeed a meaningful impact of this delay on their data. Would the group-level results change if only results from short-delays were used ?*

**Reply:** this is an important point that was also brought forward by reviewer 1. We do not think that the group-level results would change in case only short-delay data was used. However, we decided to be a bit more modest with our conclusions. See our response to reviewer 1 for more details on what we changed.

---

Second decision letter

MS ID#: bio.062674R1

MS Title: Correlating relative light sensitivity to retinal visual gene expression patterns in three African cichlid fish species

Authors: Gerrit Potkamp; Elena Zhemchuzhnikova; Felicity van Geresteijn; Joyce A. Luijben; Floor A. Munnik; Thomas A. Veldhuis; Martine E. Maan; Louis van de Zande; Rampal S. Etienne

© 2026. Published by The Company of Biologists under the terms of the Creative Commons Attribution License (https://creativecommons.org/licenses/by/4.0/).

I am happy to tell you that your manuscript has been accepted for publication in Biology Open, pending our standard publication integrity checks. It was accepted on 4th September 2026.

© 2026. Published by The Company of Biologists under the terms of the Creative Commons Attribution License (https://creativecommons.org/licenses/by/4.0/).
